# Diet and Risk of Gastric Cancer: An Umbrella Review

**DOI:** 10.3390/nu14091764

**Published:** 2022-04-23

**Authors:** Emmanouil Bouras, Konstantinos K. Tsilidis, Marianthi Triggi, Antonios Siargkas, Michail Chourdakis, Anna-Bettina Haidich

**Affiliations:** 1 Department of Hygiene, Social & Preventive Medicine and Medical Statistics, School of Medicine, Faculty of Health Sciences, Aristotle University of Thessaloniki, 54124 Thessaloniki, Greece; ebouras@gapps.auth.gr (E.B.); antonis.siargkas@gmail.com (A.S.); mhourd@gapps.auth.gr (M.C.); 2 Department of Hygiene and Epidemiology, University of Ioannina School of Medicine, 45110 Ioannina, Greece; marianthitrigki@gmail.com; 3 Department of Epidemiology and Biostatistics, School of Public Health, Imperial College London, London SW7 2BX, UK

**Keywords:** diet, nutrition, risk factors, gastric cancer, stomach cancer, umbrella review

## Abstract

Several dietary exposures have been associated with gastric cancer (GC), but the associations are often heterogenous and may be afflicted by inherent biases. In the context of an Umbrella Review (UR), we provide an overview and a critical evaluation of the strength and quality, and evidence classification of the associations of diet-related exposures in relation to the risk of GC. We searched PubMed and Scopus for eligible meta-analyses of observational studies published in English from inception to 12 December 2021, and for any identified association, we applied robust epidemiological validity evaluation criteria and individual study quality assessment using AMSTAR. We screened 3846 titles/abstracts and assessed 501 full articles for eligibility, of which 49 were included in the analysis, investigating 147 unique exposures in relation to GC, cardia (GCC) or non-cardia (GNCC) cancer. Supported by suggestive evidence, positive associations were found comparing the highest vs. lowest categories for: heavy (>42 g/day) alcohol consumption (Relative Risk (RR) = 1.42, 95% Confidence Interval (CI): 1.20–1.67), salted fish consumption (RR = 1.56, 95% CI:1.30–1.87) and waist circumference (RR = 1.48, 95% CI:1.24–1.78) and an inverse association for the healthy lifestyle index (RR = 0.60, 95% CI:0.48–0.74) in relation to GC. Additionally, a positive association was found comparing obese individuals (Body Mass Index (BMI) ≥ 30) to normal-weight individuals (BMI: 18.5–25) (RR = 1.82, 95% CI:1.32–2.49) in relation to GCC. Most of the meta-analyses were of medium-to-high quality (median items: 7.0, interquartile range: 6–9). Maintaining a normal body weight and adopting healthy dietary choices, in particular, limiting the consumption of salt-preserved foods and alcohol, can reduce the risk of gastric cancer.

## 1. Introduction

Gastric cancer (GC), also known as stomach cancer, is among the five most common cancers worldwide, accounting for more than 750,000 deaths in 2020 [1]. Most of the new cases occur in areas with lower socio-economic indices, with approximately half of the newly diagnosed cases located in East Asia. Incidence rates are twice as high in men than in women and are increasing with age for both sexes [1]. From a histopathological perspective, approximately 90% of gastric cancers are adenocarcinomas [2]. Gastric cancers are typically classified based on their anatomical location into cardia (arising in the area of the stomach proximal to the gastroesophageal junction) and non-cardia, with the latter being the most common of the two [3].

The global rates of gastric non-cardia cancer (GNCC) are declining, which can be attributed to a lower prevalence of Helicobacter pylori infection (H. pylori), a well-established risk factor for GNCC [4]. On the other hand, gastric cardia cancer (GCC) incidence rates are increasing, which is consistent with the global increase in obesity and adaptation to a western lifestyle [5]. Other established exposures associated with an increased GC risk include tobacco use and industrial and chemical pollutants (e.g., wood processing, coal mining, rubber manufacturing and chromium IV) [6]. Diet-related exposures that have been associated with an increased GC risk include body fatness (GCC), alcohol consumption (based on evidence for intakes above 45 g/day) and salt-preserved foods [7]. Several other diet-related exposures have been associated with GC, but results are often conflicting [8].

The aim of the present study is to provide an overview and a critical evaluation of the strength and quality, and classification of the existing epidemiological evidence that investigates the association of diet-related exposures in relation to risk of GC, through the prism of an umbrella review, and, where possible, to highlight methodological gaps and reasons for inconsistency.

## 2. Materials and Methods

This umbrella review follows the proposed reporting standards, using publicly available checklists [9,10]. Furthermore, the study protocol was registered at open science framework (https://osf.io/pd5br/).

### 2.1. Study Selection and Data Extraction

Two reviewers independently searched MEDLINE via PubMed and Elsevier’s Scopus for eligible meta-analyses published in English from inception to 12 December 2021 using diet- and gastric-cancer-related terms (Table S1).

Eligible for inclusion were meta-analyses that examined the association between any diet-related exposure in relation to GC risk. No restrictions were made regarding population characteristics, such as age, gender, ethnicity or setting. Studies that only examined survival or recurrence, or GC mortality (with no consideration of first onset GC risk endpoints), and narrative reviews were excluded from the review.

Title, abstract and full text screening was performed independently by two reviewers (EB and AS), using a publicly available systematic review tool (Rayyan), and potential discrepancies were resolved by a third reviewer (ABH) [11]. In the case that more than one meta-analysis was identified for an association, only the most recent or complete study (the one providing all the defined items to extract) was selected for the analyses, provided the study’s quality was sufficient.

Data were extracted by two reviewers (AS and MT) using a predefined data extraction form and verified by another reviewer (EB). From each meta-analysis, the following items were extracted: definition of exposure and type of contrast (i.e., continuous exposure or comparing top versus bottom exposure categories), sample size (number of cases and controls), effect size of the association (i.e., relative risk (RR), along with 95% confidence intervals (CI)) and design of each primary study.

### 2.2. Statistical Analyses

Where available, analyses were performed separately using primary studies with a prospective design and case–control studies. In the case that both the prospective and case–control studies were available for a given association, the prospective ones were included in the main analysis, and the case–control one were analyzed separately in a sensitivity analysis.

The effect size of each association was estimated using a random effects meta-analysis, by means of RR along with 95% CI [12]. The inconsistency index (Ι^2^) was used, and the prediction intervals (PI) were estimated to further quantify the heterogeneity [13,14]. Small study effects were investigated using Egger’s regression and by applying an excess significance test [15,16]. Summary statistics were presented as a mean and standard deviation (SD) or median and interquartile range (IQR), and frequencies with percentages.

The description of the included studies in the present umbrella review was also supported using describing bibliographic information as provided by Scopus, such as paper author, journals, affiliated countries and citations [17].

All statistical analyses were performed using the libraries meta, metafor & bibliometrix in R v. 4.1.0. [17,18,19,20].

### 2.3. Evidence Classification and Quality Assessment

Each unique association that was nominally significant (*p*-value < 0.05) was classified as strong evidence (Class I), highly suggestive evidence (Class II), suggestive evidence (Class III) or weak associations (Class IV), with regard to epidemiological validity criteria (Table S2) [21]. In brief, to consider that an association is supported by strong evidence, all of the following criteria should be met: inclusion of >1000 cancer cases; random-effects MA *p*-value ≤ 10^−6^; absence of high heterogeneity (I^2^ < 50%); 95% prediction intervals excluding the null; and no evidence of small study effects and excess significance bias. Furthermore, the quality of the included studies was evaluated using AMSTAR [22].

Biases, such as selection and information bias, are commonly distorting the associations in observational studies, resulting in larger estimates with small *p*-values. To minimize the extent to which pertinent biases might affect the results of our study, the main analysis was based on prospective evidence only (cohort studies). Separate analyses were performed including both prospective and non-prospective (case–control) studies.

## 3. Results

### 3.1. Overview of the Included Studies

**Figure 1:**
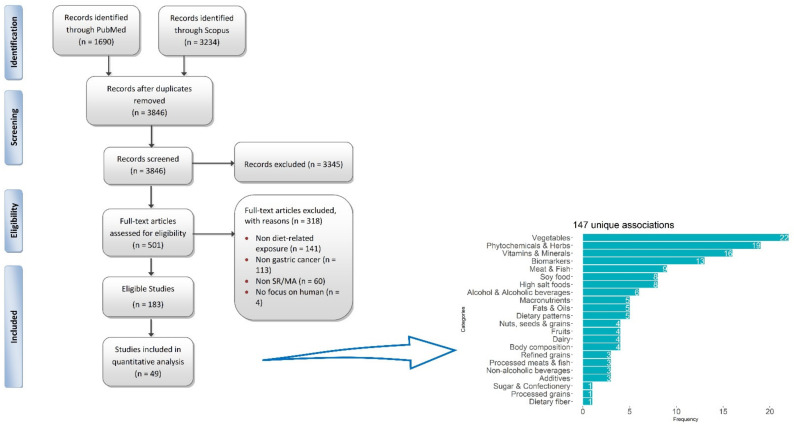
Flow chart of the study selection process (**left**) and overview of the dietary exposures that are evaluated in the present umbrella review, by category (**right**).

The literature search yielded 3846 unique articles, of which 3345 were excluded during the title and abstract screening and a further 318 were excluded during the full-text screening. Of the 183 eligible studies, 49 systematic reviews with MA examining 147 unique exposures in relation to GC, GCC or GNCC were finally included in the umbrella review (Figure 1). The 49 included studies were published between 2008 and 2021, in 35 scientific journals, with an average of 5.8 years elapsed from their publication until the date of the last literature search. The median number of authors per study was 5 (range: 2–43), none was single authored and the average number of citations per study per year was 5.5 (range: 0–40). China was the most frequent (61.2%; 30/49) corresponding author’s country (Figure S1). Most of the 147 exposures belong to the vegetable family (n = 22), followed by exposures pertaining to phytochemicals (n = 19), micronutrients (n = 16), biomarkers (n = 13) and meat products (n = 9) (a categorization is shown in Figure 1).

The majority of the exposures (78.2%; 115/147), for which prospective primary data were available, were evaluated in relation to GC in the classification process; 15 were evaluated in relation to GCC and 14 in relation to GNCC (Table S3). Sensitivity analysis, including both prospective and non-prospective studies, was performed for all the 147 exposures in relation to GC, 23 in relation to GCC and 22 in relation to GNCC (Table S4).

### 3.2. Gastric Cancer

**Figure 2:**
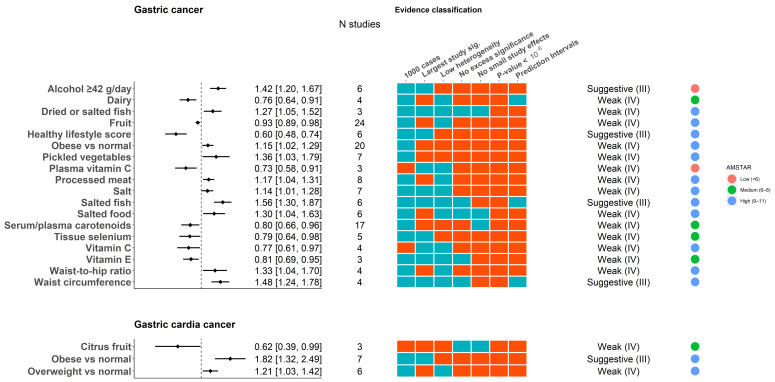
Summary of the significant associations (*p*-value < 0.05) between the dietary exposures and gastric and gastric non-cardia cancer, and the classification of the evidence using epidemiological validity criteria. The forest plot shows the random effect meta-analysis relative risks and the 95% confidence intervals. The heatmap shows the epidemiological validity criteria that are fulfilled per association (blue box indicates ‘yes’; red box indicates ‘no’). Bubble column to the right of the plot summarizes the study quality based on AMSTAR. Associations that were based on >2 studies are plotted.

The 115 associations with GC were based on a median of 3 primary studies (range: 2–24), 1586 (258–145,701) cases and 642,876 (2672–25,216,666) total participants. Under a random effects MA, 24 of 115 associations (20.9%) were nominally significant (*p*-value < 0.05): 2 (1.7%) had *p*-values less than 10^−6^; 86 (74.8%) were based on more than 1000 cases; 85 (73.9%) had low heterogeneity (I2 < 50%); 3 (2.6%) had significant prediction intervals; 83 (72.2%) did not have evidence of small study effects; and 88 (76.5%) had no evidence of excess significance bias, yielding no strong or highly suggestive associations (Classes I and II), but 4 were suggestive evidence (Class III) (Figure 2; Table S3). In particular, positive associations were found for high alcohol consumption (heavy drinking (>42 g/day) vs. light/non-drinking, RR = 1.42, 95% CI: 1.20–1.67), salted fish consumption (highest vs. lowest categories, RR = 1.56, 95% CI: 1.30–1.87) and waist circumference (highest vs. lowest categories, RR = 1.48, 95% CI:1.24–1.78) and an inverse association for the healthy lifestyle score, which incorporates healthy dietary choices and lifestyle factors, such as smoking abstinence and physical activity (highest vs. lowest categories, RR = 0.60, 0.48–0.74), supported by suggestive evidence (Figure 2; Table S3). A minimum set of adjustment variables (including any two of the typically reported in the studies covariates: age, sex, smoking, body mass index (BMI) and alcohol) was applied in 0/6 primary studies in the analysis for heavy alcohol drinking, 6/6 for salted fish, 4/4 for waist circumference and 6/6 for healthy lifestyle score, and only one primary study in these associations reported adjustment for H. pylori infection.

### 3.3. Gastric Cardia and Non-Cardia Cancer

The 15 associations with GCC were based on a median of 2 primary studies (range: 2–7), 547 (96–3666) cases and 203,437 (5702–2,073,362) participants. Under a random effects MA, less than a third (4/15) of the associations were nominally significant, while no association had *p*-value less than 10–6; only 2 were based on more than 1000 cases; most (10/15) had low heterogeneity, but no association had significant prediction intervals; less than half (6/15) had no evidence of small study effects and almost all (14/15) had no evidence of excess significance bias. Only one association, comparing obese individuals (with BMI ≥ 30 kg/m^2^) to normal-weight individuals (BMI: 18.5–25), was supported with suggestive evidence (RR = 1.82, 95%CI: 1.32–2.49) (Figure 2; Table S3), for which most (6/7) primary studies reported adjustment for any two of the age, sex, smoking, BMI and alcohol.

The 14 associations with GNCC were based on a median of 3 primary studies (range: 2–8), 925 (192–8391) cases and 438,515 (95,402–3,774,380) participants. Only one nominal association was found, but it was based on just two primary studies, hence providing little evidence for an association (Table S3).

### 3.4. Sensitivity Analysis

In a separate analysis that included both prospective and case–control primary studies, 58/147, 7/23 and 6/22 were nominally significant (*p*-value < 0.05) for GC, GCC and GNCC, respectively, under a random effects MA (Table S4). There were no strong (Class I) associations, and there was one highly suggestive association (Class II) for vitamin C intake in relation to GC (highest vs. lowest categories, RR = 0.58, 95% CI: 0.51–0.65). Positive suggestive associations were found for alcohol, heavy alcohol (≥42 g/day), chili, nitrites, red and processed meat, refined grains, salted fish and waist circumference, and inverse suggestive associations for b-carotene, cabbage, carotenoid, cruciferous vegetables, dietary fiber, garlic, healthy lifestyle score, Mediterranean Diet Score, onion, total soy food, vegetable fat, vitamins A and E and whole grains in relation to GC. Furthermore, a positive suggestive association was found in the analysis comparing obese individuals (BMI ≥ 30) to normal-weight individuals (BMI:18.5–25) in relation to GCC, and suggestive positive associations for red and processed meat and total fat intake, in relation to GNCC (Table S4).

Comparing the main and the sensitivity analyses, only 13 associations were qualitatively discrepant in the evidence judgement for GC. These regarded allium vegetables, b-carotene, cabbage, dietary fiber, onion, tofu, total soy food, vitamin B6 and whole grains (which were classified as non-significant based on prospective studies in contrast to an inverse association that was found in sensitivity analyses), alcohol, nitrites and red meat (non-significant association based on prospective studies contrarily to a positive association in the sensitivity analysis) and dairy for which a nominal inverse association (Class IV) was found based on prospective studies, but was non-significant in the sensitivity analysis (Figure S2).

### 3.5. Quality Assessment

**Figure 3:**
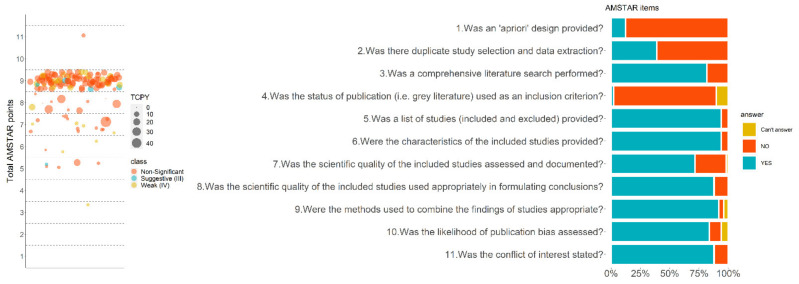
Quality assessment of the included studies using AMSTAR. In the bubble plot (**left**), each bubble represents an association that is colored based on the classification output and the size is analogous to the number of citations per year that the study received. The bar plot (**right**) provides a summary of the assessment, per item, across the 48 studies.

Quality assessment using AMSTAR showed that most of the studies were of medium to high quality (median item score = 7.0, IQR: 6–9). All the AMSTAR items were fulfilled in at least 70% of the included studies except for item 1 (presence of “a priori” protocol, 12.2%; 6/49), item 2 (duplicate study selection and data extraction, 40.8%; 20/49) and item 4 (search for grey literature, 2.0%; 1/49) (Figure 3 and Table S5).

## 4. Discussion

Our analysis confirms a positive association for highly salted fish consumption, heavy alcohol drinking (>42 g/day) and abdominal obesity and an inverse association for a healthy lifestyle index, which includes healthy dietary choices in combination with other lifestyle factors (such as smoking abstinence and physical activity) in relation to the risk of GC.

High salt consumption, and particularly the high consumption of salt-preserved foods, such as fish, vegetables and meats, emerged as a probable GC risk factor in the 1970s [23]. Ever since, several epidemiological studies have consistently reported positive associations with GC [24,25,26]. The World Cancer Research Fund (WCRF) Continuous Update Program (CUP) in its 2018 report observed a 15% increased GC risk in the analysis comparing the highest versus lowest categories of salted fish consumption, with little evidence, though, for a dose–response effect, and a 9% increased risk per 20 g of pickled vegetable consumption [7]. Our results are in line with these observations, suggesting that high salted fish intake was associated with an increased GC risk, while salt and several other high salt content exposures, such as salted fish products and pickled vegetables, showed nominal associations. It should be noted, though, that the reliable quantification of total dietary salt intake is challenging and often prone to measurement errors in a typical epidemiological setting [27]. The mechanisms underlying the observed associations are complex and implicate more than sodium chloride per se. The high consumption of salt preserved foods may be an indicator of poor diet quality and low socio-economic status, which might increase susceptibility to H. pylori infection and hence increase GC risk [7]. Furthermore, the high nitrate and nitrite content that is often found in these foods promotes the formation of N-nitroso compounds with carcinogenic properties [28]. A habitual high salt intake can also be harmful to the gastric mucosa, leading to chronic inflammation and glandular atrophy, increasing DNA damage and cell proliferation and susceptibility to H. pylori infection [29].

Increased body fatness has been associated with 13 distinct types of cancer, including GCC, demonstrating the fact that it has both systemic and tissue-specific, cancer promoting effects [30]. Obesity is conducive to chronic low-grade inflammation, hyperinsulinemia, hyperleptinemia and an elevated production of endogenous sex steroid hormones, all of which may contribute to tumor growth [31]. Furthermore, the accumulation of peri-abdominal fat increases the intra-abdominal pressure, which may in turn lead to gastroesophageal reflux and the formation of precancerous lesions in the stomach [32]. A 32% increased GCC risk per 5 kg/m^2^ was found in a dose–response MA of seven prospective studies and a similar association was identified in the WCRF 2018 report, with no evidence of association for GNCC [7]. In line with prior research, our findings suggest that obesity, particularly abdominal obesity as measured by waist circumference, is positively associated with GCC risk.

A 42% increased GC risk, supported by suggestive evidence, was found in our analysis for alcohol consumption at 42 g/day or higher, but no association was found, based on prospective evidence, for moderate habitual consumption in comparison to non-drinking. In a similar fashion, a significant linear dose–response association has been previously reported, only at levels of 45 g of ethanol per day and above [7]. The observed association finds high mechanistic support, as alcohol may act as a solvent in the stomach, enabling the penetration of substances with carcinogenic properties into the gastric cells, and interferes with prostaglandin production and retinoid metabolism [33]. In addition, alcohol may increase the production of biomolecule-toxic free radicals, while acetaldehyde, an alcohol metabolite, has been classified as a class 1 human carcinogen by the International Agency for Research on Cancer (IARC) [34]. It should be noted though that most of the primary study estimates for the above association were minimally adjusted.

Previous studies have shown that the high consumption of fruit is associated with a decreased GC risk. Specifically, a 5% decreased GC and a 24% decreased GCC risk, per 100 g of fruit and citrus fruit consumption per day, respectively, have been previously reported [7,35]. The possibility, though, that the observed inverse association for fruit consumption may be due to residual confounding by smoking (or H. pylori infection) cannot be ruled out; nevertheless, probable mechanisms to support the observed associations have been described [36]. Fruits, and in particular citrus fruits, have high concentrations of vitamin C, carotenoids and other phytochemicals (such as naringenin) and antioxidants, which protect against oxidative damage [37]. Vitamin C has the ability to regenerate other antioxidant molecules (such as vitamin E) and to inhibit nitrosamine formation in the stomach, thus conferring protection to the gastric mucosa [38]. We only found weak inverse associations for fruit in relation to GC and citrus fruit in relation to GCC, in line with previous reports [7]. Accumulating evidence suggests that free radical overproduction and/or inability to effectively neutralize them, may promote chronic inflammation, a well-established cancer enabling characteristic, via Nuclear Factor kappa b (NF-kB), STAT3 and other molecular pathways [39,40]. Considering that nominally significant (but weak) inverse associations for vitamin C and E, plasma carotenoid and tissue selenium, based on prospective evidence, and several other exposures with antioxidant and anti-inflammatory properties were found, and considering the mechanist plausibility for a protective effect in relation to GC, further investigation of these dietary exposures as a potential preventative measure is warranted.

A combination of healthy lifestyle factors, captured by pertinent scales, was associated with a suggestive inverse association with GC. Diverse scoring systems were utilized in the primary studies, which included heterogenous populations, with different socio-economic backgrounds [41,42,43]. These indices included (a combination of) smoking, physical activity, BMI, alcohol consumption and diet-related preference patterns (such as fruit and vegetables, whole grains, red and processed meats, or a pattern-based dietary score, such as a Mediterranean Diet or the Chinese Food Pagoda score). It should be noted, though, that the observed association is probably attributed to the effect of smoking, which is the strongest contributor among all variables in the lifestyle index. Stratified analyses using studies that did not include smoking in the lifestyle scores (but adjusted for smoking status) still provided statistically significant estimates for overall cancer risk, but a similar observation was not made for GC (based on 2 studies, approximately 900 cases) [44]. It is also possible that diet-related exposures (such as salt-rich foods, high alcohol consumption and excess body fatness) may act in synergy to increase the risk of GC [44].

We provide a comprehensive analysis of a wide range of dietary exposures, using a rigorous evidence credibility evaluation methodology. Furthermore, by focusing on prospectively collected data from well-established cohort studies as our primary unit of analysis, we limit the extent to which the observed associations may be afflicted by biases, such as selection and information bias. Additionally, the large sample size that most of the studies were based on provides enough power to identify emerging associations. On the other hand, our study has several limitations, most of which stem from the primary studies: it lacks an evaluation of the linear or dose–response associations, and an evaluation of the associations by sex. Furthermore, dietary assessment using non-validated approaches, inadequate adjustments in some of the primary studies, residual confounding and the absence of follow-up dietary assessments to capture potential changes in the participants’ habits are some of the inherent limitations. It should be noted that H. pylori infection was not considered in the vast majority of the primary studies. A widespread testing for and eradication of H. pylori infection is not supported; however, gaining a better understanding of the determinants of host susceptibility to GC, post infection, is important to inform prevention strategies. Estimates across meta-analysis were not pooled in the present umbrella review; hence, primary study overlapping is not considered a source of bias. Nevertheless, participant overlapping (e.g., participants from one cohort contributing to multiple associations) may have occurred to an extent, inducing a correlation of the MA estimates, but this is unlikely to have biased the associations substantially.

## 5. Conclusions

In conclusion, maintaining a normal body weight and adopting healthy dietary choices, in particular, limiting consumption of salt-preserved foods and alcohol, in combination with other lifestyle factors, such as smoking abstinence and regular physical activity, reduce the risk of developing gastric cancer.

## Data Availability

All data used in this work are presented in the supplement and are available in the original publications.

## Supplementary Materials

The following supporting information can be downloaded at: https://www.mdpi.com/article/10.3390/nu14091764/s1, Figure S1: Summary of the bibliometric descriptive regarding country and university productivity, on the included studies; Figure S2: Discrepant evidence between main analysis (including only prospective studies) and sensitivity analysis (including both prospective and case–control studies). Only associations that were qualitatively discrepant are plotted; Table S1: Search strategies that were used for the PubMed and Scopus literature searches; Table S2: Epidemiological validity criteria that were used to classify the evidence into four categories; Table S3: Evidence classification of the associations between diet and gastric cancer and subtypes based on prospective studies; Table S4: Associations between diet and gastric cancer and subtypes from the sensitivity analysis, including both prospective and case–control primary studies. Table S5: AMSTAR classification. These are the references of the 49 studies [35,44,45,46,47,48,49,50,51,52,53,54,55,56,57,58,59,60,61,62,63,64,65,66,67,68,69,70,71,72,73,74,75,76,77,78,79,80,81,82,83,84,85,86,87,88,89,90,91] that have been included in our Umbrella Review.
