# Supplementary material for: Diet and Risk of Gastric Cancer: An Umbrella Review
Source: Nutrients. 2022 Apr 23;14(9):1764. doi: 10.3390/nu14091764 (PMC9105055; doi:10.3390/nu14091764)
Supplement: Supplementary file 1 [file nutrients-14-01764-s001.zip › nutrients-1671709-supplementary.pdf]

Figure S1. Summary of the bibliometric descriptive regarding country and university productivity, on the included studies.

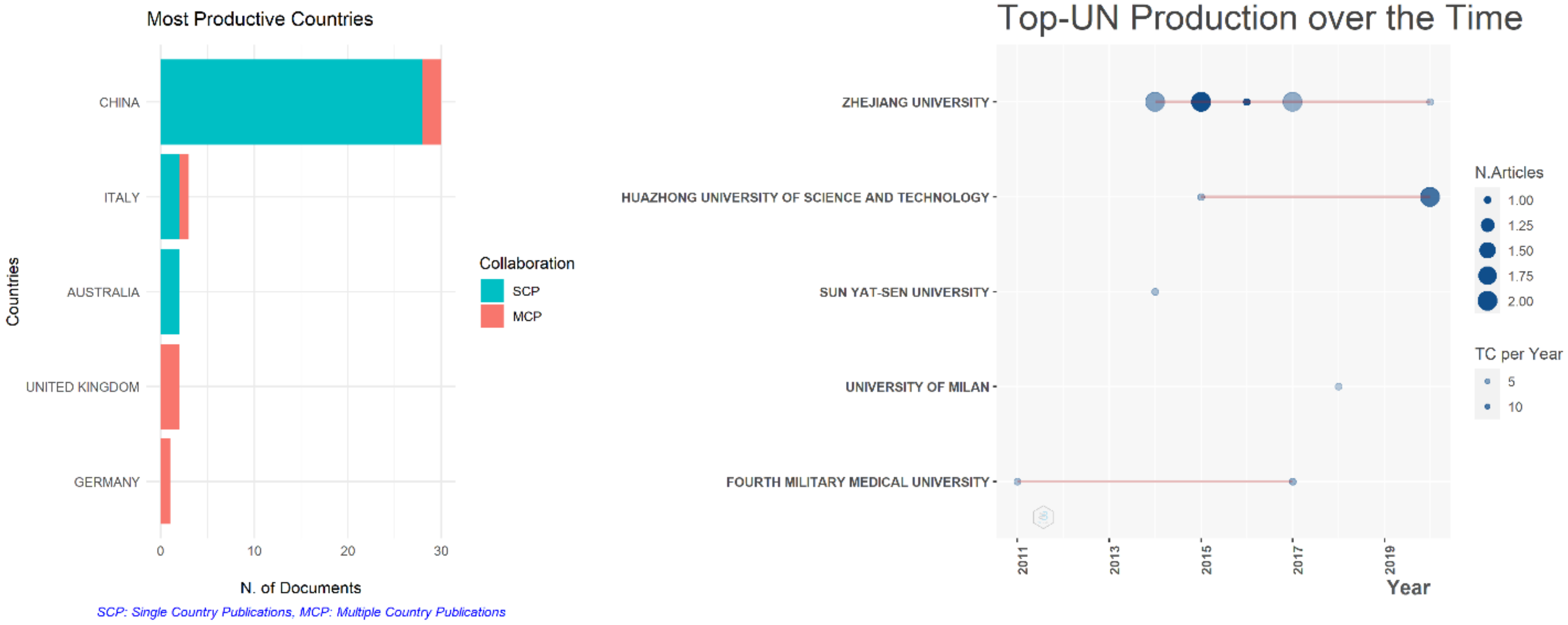

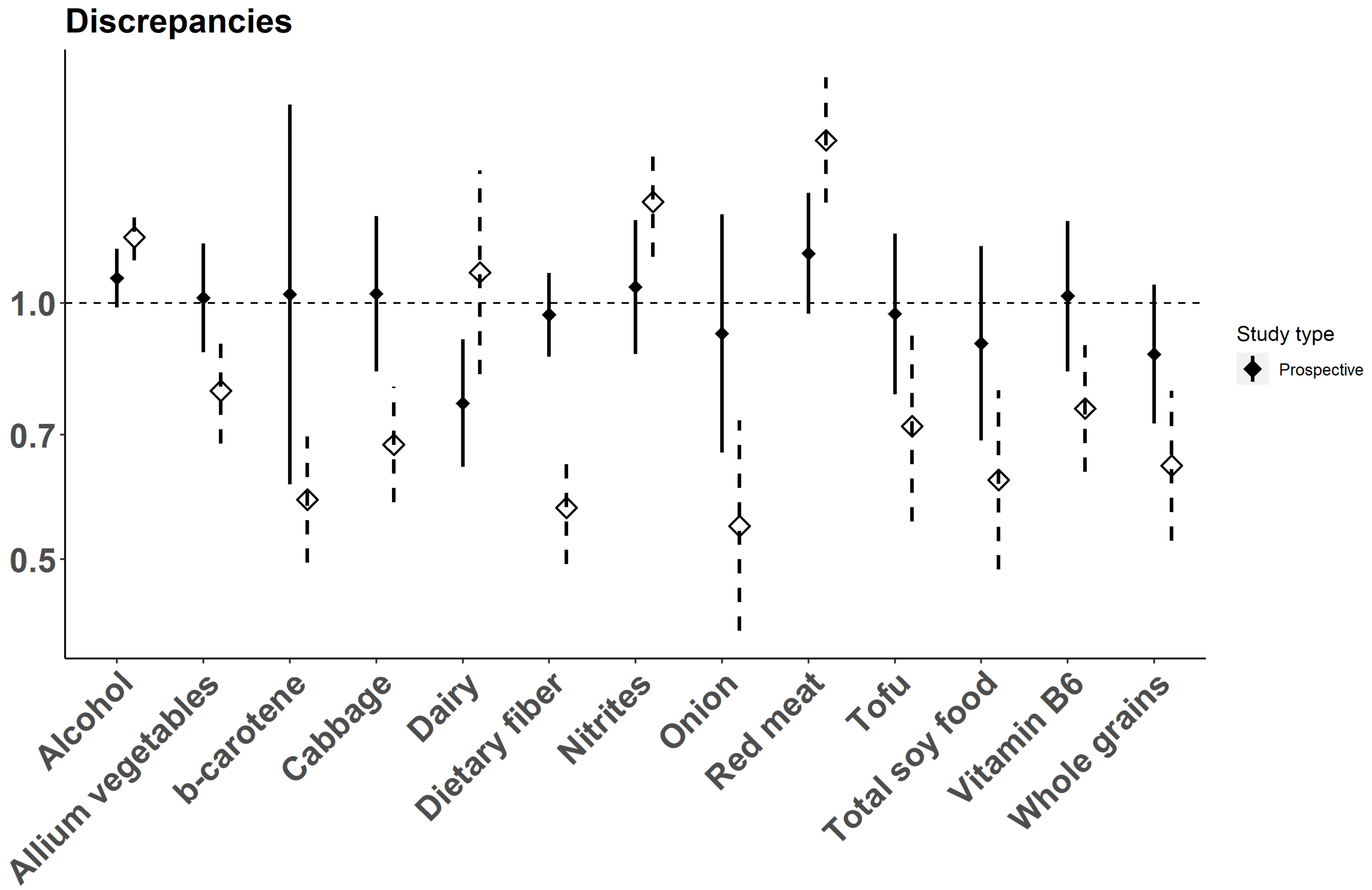

Figure S2. Discrepant evidence between main analysis (including only prospective studies) and sensitivity analysis (including both prospective and case-control studies). Only associations that were qualitatively discrepant are plotted.

Table S1. Search strategies that were used for the PubMed and Scopus literature searches.

**PubMed**
((((((((((((Stomach neoplasms[MeSH Terms]) OR (Stomach neoplasm*[tiab] OR stomach cancer*[tiab] OR stomach carcino* OR stomach tumo*[tiab] OR stomach metasta* [tiab] OR stomach malign*[tiab] OR stomach adenocarcinoma*[tiab])) OR (Gastric neoplasm* [tiab] OR gastric cancer*[tiab] OR gastric carcino* [tiab] or gastric tumo*[tiab] OR gastric metasta*[tiab] OR gastric malign*[tiab] OR gastric adenocarcinoma* [tiab])) OR (Gastrointestinal neoplasms[mesh terms] OR gastrointestinal neoplas*[tiab] OR gastrointestinal cancer*[tiab] OR gastrointestinal carcino*[tiab] OR gastrointestinal tumo*[tiab] OR gastrointestinal metasta*[tiab] OR gastrointestinal malign*[tiab] OR gastrointestinal adenocarcinoma*[tiab])) OR (Digestive tract neoplasm*[tiab] OR digestive tract cancer*[tiab] OR digestive tract carcino*[tiab] OR digestive tract tumo*[tiab] OR digestive tract metasta*[tiab] OR digestive tract malign*[tiab] OR digestive tract adenocarcinoma*[tiab])) OR (Alimentary tract neoplasm*[tiab] OR alimentary tract cancer*[tiab] OR alimentary tract carcino*[tiab] OR alimentary tract tumo*[tiab] OR alimentary tract metasta*[tiab] OR alimentary tract malign* OR alimentary tract adenocarcinoma*[tiab])) OR (Esophagogastric neoplasm*[tiab] OR esophagogastric cancer*[tiab] OR esophagogastric carcino* OR esophagogastric tumo*[tiab] OR esophagogastric metasta* [tiab] OR esophagogastric malign*[tiab] OR esophagogastric adenocarcinoma* [tiab] OR esophagogastric neoplasm*[tiab])) OR (Esophago gastric cancer*[tiab] OR esophago gastric carcino* OR esophago gastric tumo*[tiab] OR esophago gastric metasta* [tiab] OR esophago gastric malign*[tiab] OR esophago gastric adenocarcinoma* [tiab])) OR (Oesophagogastric neoplasm*[tiab] OR oesophagogastric cancer*[tiab] OR oesophagogastric carcino* OR oesophagogastric tumo*[tiab] OR oesophagogastric metasta* [tiab] OR oesophagogastric malign*[tiab] OR oesophagogastric adenocarcinoma* [tiab])) OR (Oesophago gastric neoplasm*[tiab] OR oesophago gastric cancer*[tiab] OR oesophago gastric carcino* OR oesophago gastric tumo*[tiab] OR oesophago gastric metasta* [tiab] OR oesophago gastric malign*[tiab] OR oesophago gastric adenocarcinoma* [tiab])) OR (Stomach adenoma*[tiab] OR gastric adenoma*[tiab] OR gastrointestinal adenoma*[tiab] OR digestive tract adenoma*[tiab] OR alimentary tract adenoma*[tiab] OR esophagogastric adenoma*[tiab] OR esophagogastric adenoma*[tiab] OR oesophagogastric adenoma*[tiab] OR oesophagogastric adenoma*[tiab])))) AND ((((((((((((((((((((diet therapy[MeSH Terms] OR nutrition[MeSH Terms])) OR (diet[tiab] OR diets[tiab] OR dietetic[tiab] OR dietary[tiab] OR eating[tiab] OR intake[tiab] OR nutrient*[tiab] OR nutrition[tiab] OR vegetarian*[tiab] OR vegan*[tiab] OR "seventh day adventist"[tiab] OR macrobiotic[tiab])) OR (food and beverages[MeSH Terms])) OR (food*[tiab] OR cereal*[tiab] OR grain*[tiab] OR granary[tiab] OR wholegrain[tiab] OR wholewheat[tiab] OR roots[tiab] OR plantain*[tiab] OR tuber[tiab] OR tubers[tiab] OR vegetable*[tiab] OR fruit*[tiab] OR pulses[tiab] OR beans[tiab] OR lentils[tiab] OR chickpeas[tiab] OR legume*[tiab] OR soy[tiab] OR soya[tiab] OR nut[tiab] OR nuts[tiab] OR peanut*[tiab] OR groundnut*[tiab] OR (seeds[tiab] and (diet*[tiab] OR food*[tiab])) OR meat[tiab] OR beef[tiab] OR pork[tiab] OR lamb[tiab] OR poultry[tiab] OR chicken[tiab] OR turkey[tiab] OR duck[tiab] OR fish[tiab] OR ((fat[tiab] OR fats[tiab] OR fatty[tiab]) AND (diet*[tiab] or food*[tiab] or adipose[tiab] or blood[tiab] or serum[tiab] or plasma[tiab])) OR egg[tiab] OR eggs[tiab] OR bread[tiab] OR (oils[tiab] AND and (diet*[tiab] or food*[tiab] or adipose[tiab] or blood[tiab]or serum[tiab] or plasma[tiab])) OR shellfish[tiab] OR seafood[tiab] OR sugar[tiab] OR syrup[tiab] OR dairy[tiab] OR milk[tiab] OR herbs[tiab] OR spices[tiab] OR chilli[tiab] OR chillis[tiab] OR pepper*[tiab] OR condiments[tiab] OR tomato*[tiab])) OR (fluid intake[tiab] OR water[tiab] OR drinks[tiab] OR drinking[tiab] OR tea[tiab] OR coffee[tiab] OR caffeine[tiab] OR juice[tiab] OR beer[tiab] OR spirits[tiab] OR liquor[tiab] OR wine[tiab] OR alcohol[tiab] OR alcoholic[tiab] OR beverage*[tiab] OR (ethanol[tiab] and (drink*[tiab] or intake[tiab] or consumption[tiab])) OR yerba mate[tiab] OR ilex paraguariensis[tiab])) OR (pesticides[MeSH Terms] OR fertilizers[MeSH Terms] OR "veterinary drugs"[MeSH Terms])) OR (pesticide*[tiab] OR herbicide*[tiab] OR DDT[tiab] OR fertiliser*[tiab] OR fertilizer*[tiab] OR organic[tiab] OR contaminants[tiab] OR contaminate*[tiab] OR veterinary drug*[tiab] OR polychlorinated dibenzofuran*[tiab] OR PCDF*[tiab] OR polychlorinated dibenzodioxin*[tiab] OR PCDD*[tiab] OR polychlorinated biphenyl*[tiab] OR PCB*[tiab] OR cadmium[tiab] OR arsenic[tiab] OR chlorinated hydrocarbon*[tiab] OR microbial contamination*[tiab])) OR food preservation[MeSH Terms]) OR (mycotoxin*[tiab] OR aflatoxin*[tiab] OR pickled[tiab] OR bottled[tiab] OR bottling[tiab] OR canned[tiab] OR canning[tiab] OR vacuum pack*[tiab] OR refrigerate*[tiab] OR refrigeration[tiab] OR cured[tiab] OR smoked[tiab] OR preserved[tiab] OR

preservatives[tiab] OR nitrosamine[tiab] OR hydrogenation[tiab] OR fortified[tiab] OR additive*[tiab] OR colouring*[tiab] OR coloring*[tiab] OR flavouring*[tiab] OR flavoring*[tiab] OR nitrates[tiab] OR nitrites[tiab] OR solvent[tiab] OR solvents[tiab] OR ferment*[tiab] OR processed[tiab] OR antioxidant*[tiab] OR genetic modif*[tiab] OR genetically modif*[tiab] OR vinyl chloride[tiab] OR packaging[tiab] OR labelling[tiab] OR phthalates[tiab])) OR cookery[MeSH Terms]) OR (cooking[tiab] OR cooked[tiab] OR grill[tiab] OR grilled[tiab] OR fried[tiab] OR fry[tiab] OR roast[tiab] OR bake[tiab] OR baked[tiab] OR stewing[tiab] OR stewed[tiab] OR casserol*[tiab] OR broil[tiab] OR broiled[tiab] OR boiled[tiab] OR (microwave[tiab] and (diet*[tiab] or food*[tiab])) OR microwaved[tiab] OR reheating[tiab] OR reheating[tiab] OR heating[tiab] OR re-heated[tiab] OR heated[tiab] OR poach[tiab] OR poached[tiab] OR steamed[tiab] OR barbecue*[tiab] OR chargrill*[tiab] OR heterocyclic amines[tiab] OR polycyclic aromatic hydrocarbons[tiab] OR dietary acrylamide[tiab])) OR (((carbohydrates[MeSH Terms] OR proteins[MeSH Terms]) and (diet*[tiab] or food*[tiab])) OR sweetening agents[MeSH Terms])) OR (salt[tiab] OR salting[tiab] OR salted[tiab] OR fiber[tiab] OR fibre[tiab] OR polysaccharide*[tiab] OR starch[tiab] OR starchy[tiab] OR carbohydrate*[tiab] OR lipid*[tiab] OR ((linoleic acid*[tiab] OR sterols[tiab] OR stanols[tiab]) AND (diet*[tiab] or food*[tiab] or adipose [tiab] or blood[tiab] or serum[tiab] or plasma[tiab])) OR sugar*[tiab] OR sweetener*[tiab] OR saccharin*[tiab] OR aspartame[tiab] OR acesulfame[tiab] OR cyclamates[tiab] OR maltose[tiab] OR mannitol[tiab] OR sorbitol[tiab] OR sucrose[tiab] OR xylitol[tiab] OR cholesterol[tiab] OR protein[tiab] OR proteins[tiab] OR hydrogenated dietary oils[tiab] OR hydrogenated lard[tiab] OR hydrogenated oils[tiab])) OR vitamins[MeSH Terms]) OR (supplements[tiab] OR supplement[tiab] OR vitamin*[tiab] OR retinol[tiab] OR carotenoid*[tiab] OR tocopherol[tiab] OR folate*[tiab] OR folic acid[tiab] OR methionine[tiab] OR riboflavin[tiab] OR thiamine[tiab] OR niacin[tiab] OR pyridoxine[tiab] OR cobalamin[tiab] OR mineral*[tiab] OR (sodium[tiab] AND (diet*[tiab] or food*[tiab])) OR iron[tiab] OR ((calcium[tiab] AND (diet*[tiab] or food*[tiab] or supplement*[tiab])) OR selenium[tiab] OR (iodine[tiab] AND and (diet*[tiab] or food*[tiab] or supplement*[tiab] or deficiency)) OR magnesium[tiab] OR potassium[tiab] OR zinc[tiab] OR copper[tiab] OR phosphorus[tiab] OR manganese[tiab] OR chromium[tiab] OR phytochemical[tiab] OR allium[tiab] OR isothiocyanate*[tiab] OR glucosinolate*[tiab] OR indoles[tiab] OR polyphenol*[tiab] OR phytoestrogen*[tiab] OR genistein[tiab] OR saponin*[tiab] OR coumarin*[tiab] OR lycopene[tiab])) OR (physical fitness[MeSH Terms] OR physical exertion[MeSH Terms] OR physical endurance[MeSH Terms] or walking[MeSH Terms])) OR (recreational activit*[tiab] OR household activit*[tiab] OR occupational activit*[tiab] OR physical activit*[tiab] OR physical inactivit*[tiab] OR exercise[tiab] OR exercising[tiab] OR energy intake[tiab] OR energy expenditure[tiab] OR energy balance[tiab] OR energy density[tiab])) OR (body weight [MeSH Terms] OR anthropometry[MeSH Terms] OR body composition[MeSH Terms] OR body constitution[MeSH Terms] OR obesity [MeSH Terms] OR obesity [MeSH Terms])) OR (weight loss[tiab] or weight gain[tiab] OR anthropometry[tiab] OR birth weight[tiab] OR birthweight[tiab] OR birth-weight[tiab] OR child development[tiab] OR height[tiab] OR body composition[tiab] OR body mass[tiab] OR BMI[tiab] OR obesity[tiab] OR obese[tiab] OR overweight[tiab] OR over-weight[tiab] OR over weight[tiab] OR skinfold measurement*[tiab] OR skinfold thickness[tiab] OR DEXA[tiab] OR bio-impedence[tiab] OR waist circumference[tiab] OR hip circumference[tiab] OR waist hip ratio*[tiab] OR weight change [tiab] OR adiposity [tiab] OR abdominal fat [tiab] OR body fat distribution [tiab] OR body size [tiab] OR waist-to-hip ratio [tiab])))
NOT (animal[MeSH Terms] NOT human[MeSH Terms]) AND ((systematic[sb] OR Meta-Analysis[ptyp]))) AND (eng[lang])

Scopus

TITLE-ABS-KEY(zinc OR yerba OR xylitol OR wine OR wholewheat OR wholegrain OR weight OR water OR walking OR waist OR vitamins OR vitamin OR vinyl OR veterinary drugs OR veterinary OR vegetarian OR vegetable OR vegan OR vacuum OR turkey OR tubers OR tuber OR tocopherol OR thickness OR thiamine OR tea OR syrup OR sweetening OR sweetener OR supplements OR supplement OR sugar OR sugar OR sucrose OR stewing OR stewed OR sterols OR steamed OR starchy OR starch OR stanols OR spirits OR spices OR soya OR soy OR sorbitol OR solvents OR solvent OR sodium OR smoked OR skinfold OR shellfish OR selenium OR seeds OR seafood OR saponin OR salting OR salted OR salt OR saccharin OR roots OR roast OR riboflavin OR retinol OR re-heating OR reheating OR reheated OR refrigeration OR refrigerate OR (recreational activity) OR ratio OR pyridoxine OR pulses OR proteins OR protein OR processed OR preserved OR preservatives OR preservation OR poultry OR potassium OR pork OR polysaccharide OR polyphenol OR polycyclic OR polychlorinated OR poached OR poach OR plantain OR pickled OR phytoestrogen OR phytochemical OR (physical activity) OR phthalates OR phosphorus OR pesticides OR pesticide OR pepper OR peanut OR PCDF OR PCDD OR PCB OR paraguariensis OR packaging OR pack OR over-weight OR overweight OR over OR organic OR

oils OR occupational OR obesity OR obese OR nuts OR nutrition OR nutrient OR nut OR nitrosamine OR nitrites OR nitrates OR niacin OR mycotoxin OR mineral OR milk OR microwaved OR microwave OR microbial OR methionine OR meat OR measurement OR mate OR mannitol OR manganese OR maltose OR magnesium OR macrobiotic OR liquor OR lipid OR linoleic OR lentils OR legume OR lard OR lamb OR labelling OR juice OR isothiocyanate OR iron OR iodine OR intake OR indoles OR inactivit OR ilex OR hydrogenation OR hydrogenated OR hydrocarbons OR hydrocarbon OR hip OR heterocyclic OR herbs OR herbicide OR heating OR heated OR growth OR groundnut OR grilled OR grill OR granary OR grain OR glucosinolate OR genistein OR genetically OR genetic OR fry OR fruit OR fried OR fortified OR foods OR food OR food OR folic OR folate OR fluid OR flavouring OR flavoring OR fitness OR fish OR fibre OR fiber OR fertilizers OR fertilizer OR fertiliser OR ferment OR fatty OR fats OR fat OR expenditure OR exertion OR exercising OR exercise OR ethanol OR energy OR endurance OR eggs OR egg OR eating OR duck OR drug OR drinks OR drinking OR diets OR dietetic OR dietary OR diet OR dibenzofuran OR dibenzodioxin OR DEXA OR density OR DDT OR dairy OR cyclamates OR cured OR coumarin OR copper OR cooking OR cookery OR cooked OR contamination OR contaminate OR contaminants OR constitution OR condiments OR composition OR colouring OR coloring OR coffee OR cobalamin OR circumference OR chromium OR cholesterol OR chlorinated OR chloride OR chillis OR chilli OR chickpeas OR chicken OR chargrill OR cereal OR casserol OR carotenoid OR carbohydrates OR carbohydrate OR canning OR canned OR calcium OR caffeine OR cadmium OR broiled OR broil OR breastmilk OR breastfeeding OR breastfeed OR bread OR bottling OR bottled OR boiled OR (body mass index) OR (body composition) OR BMI OR birthweight OR (birth weight) OR biphenyl OR bio-impedence OR beverages OR beverage OR beer OR beef OR beans OR barbecue OR balance OR baked OR bake OR aspartame OR arsenic OR aromatic OR antioxidant OR anthropometry OR amines OR allium OR alcoholic OR alcohol OR agents OR aflatoxin OR additive OR activit OR acid OR acid OR acesulfame) AND (TITLE-ABS-KEY(stomach OR gastric)) AND (TITLE-ABS-KEY(cancer OR malignant OR neoplasm OR neoplasms OR tumor OR tumour OR malignancy)) and TITLE-ABS-KEY(meta-analysis OR (systematic and review) OR review) AND (LIMIT-TO (LANGUAGE,"English"))

Table S2. Epidemiological validity criteria that were used to classify the evidence into four categories.

**Class I** (strong evidence)	More than 1000 cases<br>Random effects MA P-value < 10^{-6}<br>Non-significant heterogeneity ($I^2 < 50\%$)<br>95% PI excluding the null,<br>No evidence for small study effect (P-value > 0.1)<br>No evidence for excess significance bias (P-value > 0.1)
**Class II** (highly suggestive evidence)	More than 1000 cases<br>Random effects MA P-value < 10^{-6}<br>Non-significant heterogeneity ($I^2 < 50\%$)<br>Largest study in the meta-analysis was nominally statistically significant.
**Class III** (suggestive evidence)	More than 1000 cases<br>Random effects MA P-value < 10^{-3}
**Class IV** (weak evidence)	Random effects MA P-value < 0.05

Table S3. Evidence classification of the associations between diet and gastric cancer and subtypes based on prospective studies.

ID	k_studies	Total_cases	Participants	statSig0	Effect*	Pval	PI	I2	Pegger	class	TP
**Gastric cancer**											
a-carotene (Fang, 2015)	2	1345	56209	0	0.78 [0.33, 1.85]	5.8E-01		0.85		Non-Significant	9
Alcohol (Deng, 2021)	16	145701	25216666	1	1.07 [0.99, 1.16]	9.6E-02	0.82-1.4	0.79	0.071	Non-Significant	5
Alcohol ≥42 grams/day (Deng, 2021)	6	18346	2774644	1	1.42 [1.20, 1.67]	3.6E-05	0.84-2.4	0.88	0.985	Suggestive (III)	5
Allium vegetables (Turati, 2015)	4	1776	696282	0	1.01 [0.88, 1.18]	8.5E-01	0.8-1.29	0	0.614	Non-Significant	5
Anthocyanin (Yang, 2019)	2	1980	948300	0	0.95 [0.79, 1.13]	5.6E-01	0.3-3.02	0	0.303	Non-Significant	7
Antioxidant Supplements, b-carotene, vitamins A, C, E, and Se (Bjelakovic, 2008)	12	714	157300	0	1.14 [0.98, 1.33]	1.0E-01	0.95-1.36	0	0.660	Non-Significant	11
Apples and/or pears (Fang, 2015)	3	531	212839	0	1.02 [0.95, 1.10]	5.4E-01	0.79-1.33	0.66	0.704	Non-Significant	9
b-carotene (Fang, 2015)	3	1467	84672	0	1.02 [0.61, 1.71]	9.3E-01	0.12-8.89	0.69	0.643	Non-Significant	9
b-cryptoxanthin (Fang, 2015)	2	1345	56209	0	1.09 [0.78, 1.53]	6.2E-01		0		Non-Significant	9
Bacon or side pork (Fang, 2015)	2	1473	159428	0	1.51 [1.03, 2.22]	3.5E-02		0		Weak (IV)-nominal**	9
Beans (Fang, 2015)	2	519	531436	0	0.82 [0.62, 1.10]	1.9E-01	0.13-5.31	0	0.840	Non-Significant	9
Beer (Fang, 2015)	9	2766	1782148	0	1.17 [0.99, 1.37]	5.9E-02	0.83-1.64	0.24	0.291	Non-Significant	9
Black Tea (Fang, 2015)	3	1113	606374	0	1.20 [0.81, 1.77]	3.5E-01	0.42-3.46	0.39	0.822	Non-Significant	9
Blood retinol (Wu, 2015)	5	1561	33253	0	0.87 [0.71, 1.08]	2.0E-01	0.47-1.62	0.47	0.956	Non-Significant	8
Brassica vegetables (Fang, 2015)	2	328	91987	0	0.82 [0.59, 1.14]	2.4E-01	0.03-20.85	0.41	0.208	Non-Significant	9
Bread (Fang, 2015)	4	1912	676319	0	1.15 [0.90, 1.48]	2.5E-01	0.6-2.21	0.29	0.089	Non-Significant	9
Cabbage (Fang, 2015)	4	1752	1420142	0	1.03 [0.83, 1.26]	8.1E-01	0.6-1.75	0.29	0.697	Non-Significant	9
Cheese (Fang, 2015)	2	709	566381	0	1.02 [0.66, 1.58]	9.4E-01	0.39-2.67	0	0.422	Non-Significant	9
Chicken (Fang, 2015)	3	862	641323	0	1.12 [0.86, 1.46]	4.0E-01	0.73-1.72	0	0.333	Non-Significant	9
Citrus fruit (Vingeliene, 2016)	8	5446	2219490	0	0.94 [0.83, 1.05]	2.6E-01	0.69-1.27	0.47	0.387	Non-Significant	7
Coffee (Deng, 2016)	14	3484	1324559	0	1.16 [0.99, 1.35]	6.6E-02	0.8-1.68	0.27	0.290	Non-Significant	8
Cruciferous vegetables (Fang, 2015)	4	2550	1123644	0	0.99 [0.78, 1.25]	9.3E-01	0.59-1.67	0	0.999	Non-Significant	9
Dairy (Guo, 2015)	4	1210	598220	0	0.76 [0.64, 0.91]	2.2E-03	0.6-0.97	0	0.762	Weak (IV)	7
Dietary fiber (Fang, 2015)	3	672	108941	0	0.97 [0.87, 1.09]	5.9E-01	0.47-2.02	0	0.907	Non-Significant	9
Dietary Inflammatory Index (Moazzen, 2020)	2	1076	518671	0	1.39 [1.00, 1.94]	5.0E-02		0.7		Non-Significant	9
Dried or salted fish (Fang, 2015)	3	4221	1261988	1	1.27 [1.05, 1.52]	1.2E-02	0.94-1.71	0	0.037	Weak (IV)	9
Egg (Fang, 2015)	6	2254	1202042	0	1.06 [0.87, 1.28]	5.9E-01	0.62-1.79	0.59	0.008	Non-Significant	9
Fish (Fang, 2015)	14	7117	1835050	0	1.11 [0.99, 1.23]	6.9E-02	0.98-1.24	0	0.772	Non-Significant	9
Flavan-3-ols (Grosso, 2017)	2	1366	954624	0	0.71 [0.40, 1.25]	2.4E-01		0.71		Non-Significant	8
Flavanones (Grosso, 2017)	2	1366	954624	0	0.95 [0.73, 1.24]	7.2E-01		0		Non-Significant	8
Folate (Fang, 2015)	3	1438	58676	0	1.09 [0.91, 1.30]	3.7E-01	0.73-1.61	0	0.676	Non-Significant	9
Fruit (Fang, 2015)	24	10776	3805553	0	0.93 [0.89, 0.98]	4.1E-03	0.86-1.01	0.04	0.186	Weak (IV)	9
Fruiting vegetables (Fang, 2015)	3	1422	898691	0	0.94 [0.76, 1.15]	5.5E-01	0.25-3.58	0	0.266	Non-Significant	9
Garlic (Li, 2018)	2	258	6857	0	0.92 [0.60, 1.41]	7.0E-01		0.2		Non-Significant	8
Glycemic Index (Ye, 2016)	2	48574	507610	1	1.08 [0.61, 1.93]	7.9E-01		0.75		Non-Significant	7
Glycemic Load (Ye, 2016)	2	48574	507610	0	0.96 [0.61, 1.50]	8.5E-01		0.38		Non-Significant	7
Green-yellow vegetables (Fang, 2015)	3	2024	130837	0	1.05 [0.90, 1.21]	5.6E-01	0.4-2.71	0	0.796	Non-Significant	9
Green and yellow vegetable (Fang, 2015)	2	467	128002	1	1.16 [0.14, 9.65]	8.9E-01		0.91		Non-Significant	9
Green leafy vegetables (Fang, 2015)	2	1125	50334	1	0.75 [0.54, 1.05]	9.4E-02		0.12		Non-Significant	9
Green tea (Fang, 2015)	8	2038	1292801	0	1.00 [0.89, 1.14]	9.5E-01	0.75-1.35	0.31	0.711	Non-Significant	9
Green vegetables (Fang, 2015)	2	475	84397	0	0.72 [0.47, 1.10]	1.3E-01		0		Non-Significant	9
Healthy lifestyle score (Zhang, 2020)	6	2974	1181751	1	0.60 [0.48, 0.74]	4.8E-06	0.3-1.17	0.62	0.370	Suggestive (III)	9
Heme iron (Fonseca-Nunes, 2014)	2	976	784575	1	1.01 [0.58, 1.76]	9.7E-01	0-786.94	0.83	0.302	Non-Significant	5
Highly salted food (Fang, 2015)	2	1044	117942	0	2.12 [0.59, 7.62]	2.5E-01	0-15575516.55	0.88	0.714	Non-Significant	9
Isoflavones (Lu, 2017)	2	1927	115673	0	0.90 [0.73, 1.10]	2.9E-01	0.44-1.83	0.4	0.293	Non-Significant	6
Isoflavones (Wang, 2021)	3	3224	584681	0	0.89 [0.77, 1.03]	1.2E-01	0.62-1.27	0.26	0.392	Non-Significant	8
Leafy vegetables (Fang, 2015)	2	1179	869558	0	1.03 [0.81, 1.30]	8.2E-01		0		Non-Significant	9
Legumes and pulses (Fang, 2015)	4	2652	1194100	0	0.88 [0.74, 1.05]	1.5E-01	0.67-1.17	0	0.278	Non-Significant	9

Lignans (Grosso, 2017)	2	894	558982	0	0.95 [0.70, 1.27]	7.1E-01	0.14-6.35	0	0.409	Non-Significant	8
Liquor (Fang, 2015)	7	2322	1300729	0	1.11 [0.85, 1.44]	4.5E-01	0.54-2.26	0.51	0.265	Non-Significant	9
Liver (Fang, 2015)	3	991	687233	1	1.34 [0.63, 2.83]	4.5E-01	0.16-11.09	0.41	0.858	Non-Significant	9
Lutein and zeaxanthin (Fang, 2015)	2	1345	56209	0	0.99 [0.57, 1.72]	9.7E-01		0.63		Non-Significant	9
Lycopene (Fang, 2015)	3	1467	84672	0	0.88 [0.67, 1.16]	3.8E-01	0.49-1.61	0	0.927	Non-Significant	9
Margarine (Fang, 2015)	2	612	642303	0	0.75 [0.56, 1.02]	6.3E-02	0.11-5.21	0	0.895	Non-Significant	9
Meat (Fang, 2015)	11	5393	1611578	0	1.00 [0.89, 1.11]	9.5E-01	0.79-1.26	0.24	0.960	Non-Significant	9
Meat products (Fang, 2015)	8	3030	758084	0	1.00 [0.84, 1.20]	9.7E-01	0.64-1.58	0.4	0.908	Non-Significant	9
Mediterranean Diet Score (Schwingshackl, 2017)	2	1403	981415	1	0.82 [0.61, 1.10]	1.8E-01	0.04-16.08	0.49	0.335	Non-Significant	7
Milk (Fang, 2015)	5	2133	1300378	0	1.06 [0.87, 1.28]	5.8E-01	0.71-1.58	0.24	0.554	Non-Significant	9
Miso soup (Fang, 2015)	10	8501	2216989	0	1.12 [0.98, 1.28]	1.0E-01	0.82-1.52	0.31	0.832	Non-Significant	9
Mushroom (Fang, 2015)	3	976	579144	0	0.97 [0.74, 1.28]	8.4E-01	0.62-1.52	0	0.407	Non-Significant	9
NDMA (Fang, 2015)	3	1691	280280	0	1.13 [0.69, 1.86]	6.2E-01	0.01-248.31	0.6	0.867	Non-Significant	9
Nitrates (Song, 2015)	5	1653	642876	0	0.91 [0.77, 1.09]	3.2E-01	0.74-1.13	0	0.761	Non-Significant	9
Nitrites (Song, 2015)	4	1545	630861	0	1.04 [0.87, 1.25]	6.4E-01	0.73-1.5	0.2	0.243	Non-Significant	9
Non-citrus fruit (Fang, 2015)	2	1273	39445	0	0.98 [0.95, 1.02]	2.8E-01		0		Non-Significant	9
Nut consumption (Zhang, 2020)	2	2207	689466	1	0.79 [0.72, 0.86]	3.0E-08		0		Weak (IV)-nominal**	6
Obese vs normal (Chen, 2013)	20	35023	10281810	0	1.15 [1.02, 1.29]	1.9E-02	0.75-1.76	0.65	0.165	Weak (IV)	9
Onion & Garlic (Fang, 2015)	2	1179	869558	0	0.92 [0.74, 1.14]	4.3E-01		0		Non-Significant	9
Onion (Fang, 2015)	2	305	101690	0	0.92 [0.67, 1.27]	6.2E-01	0.02-34.72	0.75	0.745	Non-Significant	9
Overweight vs normal (Chen, 2013)	15	31535	6182768	0	1.01 [0.95, 1.07]	8.4E-01	0.86-1.18	0.43	0.408	Non-Significant	9
Pickled vegetables (Fang, 2015)	7	6803	970667	0	1.36 [1.03, 1.79]	3.1E-02	0.57-3.22	0.71	0.611	Weak (IV)	9
Pickles (Fang, 2015)	7	2832	1802230	0	1.09 [0.99, 1.20]	7.6E-02	0.97-1.23	0	0.783	Non-Significant	9
Plasma vitamin C (Lam, 2013)	3	902	3065	1	0.73 [0.58, 0.91]	5.7E-03	0.17-3.1	0	0.922	Weak (IV)	3
Potato (Fang, 2015)	4	1382	705477	0	0.96 [0.92, 1.00]	5.6E-02	0.91-1.02	0	0.970	Non-Significant	9
Poultry (Fang, 2015)	2	1917	604978	1	0.96 [0.39, 2.38]	9.3E-01		0.84		Non-Significant	9
Processed meat (Fang, 2015)	8	3772	1107552	0	1.17 [1.04, 1.31]	7.9E-03	0.95-1.43	0.11	0.109	Weak (IV)	9
Raw vegetables (Fang, 2015)	3	2450	222378	0	0.96 [0.77, 1.21]	7.5E-01	0.67-1.39	0	0.012	Non-Significant	9
Red meat (Zhanwei Zhao, 2017)	4	2408	744690	0	1.14 [0.97, 1.35]	1.1E-01	0.8-1.64	0	0.495	Non-Significant	9
Retinol (Fang, 2015)	4	1667	205524	1	0.76 [0.47, 1.23]	2.7E-01	0.15-3.87	0.69	0.301	Non-Significant	9
Rice (Fang, 2015)	3	2721	1043328	0	1.08 [0.89, 1.31]	4.6E-01	0.7-1.65	0	0.850	Non-Significant	9
Root vegetables (Fang, 2015)	2	775	332289	0	0.66 [0.30, 1.49]	3.2E-01		0.89		Non-Significant	9
Salt (Fang, 2015)	7	6553	2301836	1	1.14 [1.01, 1.28]	3.5E-02	0.88-1.47	0.24	0.310	Weak (IV)	9
Salted fish (Fang, 2015)	6	5022	1039934	1	1.56 [1.30, 1.87]	1.4E-06	1.24-1.96	0.01	0.572	Suggestive (III)	9
Salted fish roe (Fang, 2015)	2	3891	740537	1	1.68 [1.37, 2.06]	7.9E-07	0.44-6.38	0	0.682	Weak (IV)	9
Salted food (Fang, 2015)	6	3128	1673808	0	1.30 [1.04, 1.63]	2.1E-02	0.98-1.73	0	0.042	Weak (IV)	9
Sausage (Fang, 2015)	2	1600	560027	0	1.49 [1.10, 2.02]	9.6E-03	0.21-10.7	0	0.278	Weak (IV)	9
Seaweed (Fang, 2015)	5	2197	1192289	0	1.02 [0.83, 1.25]	8.7E-01	0.78-1.33	0	0.461	Non-Significant	9
Serum lycopene (Yang, 2013)	3	946	2672	0	0.78 [0.56, 1.09]	1.5E-01	0.05-12.35	0.2	0.175	Non-Significant	5
Serum Vitamin D (Khayatzadeh, 2015)	2	512	50407	0	1.02 [0.76, 1.37]	8.9E-01	0.12-8.65	0.07	0.424	Non-Significant	8
Serum/plasma a-carotene (Zhou, 2016)	3	946	20346	0	0.79 [0.47, 1.31]	3.5E-01	0-147.89	0.53	0.688	Non-Significant	7
Serum/plasma b-carotene (Zhou, 2016)	7	1952	93512	0	0.72 [0.49, 1.06]	9.4E-02	0.22-2.38	0.69	0.679	Non-Significant	7
Serum/plasma carotenoids (Zhou, 2016)	17	1952	93512	0	0.80 [0.66, 0.96]	1.8E-02	0.43-1.49	0.53	0.065	Weak (IV)	7
Serum/plasma lutein (Zhou, 2016)	4	1428	21881	0	0.97 [0.76, 1.26]	8.4E-01	0.44-2.15	0.25	0.310	Non-Significant	7
Serum/plasma lycopene (Zhou, 2016)	3	946	20346	0	0.78 [0.56, 1.09]	1.5E-01	0.05-12.35	0.2	0.175	Non-Significant	7
Soy bean (Wang, 2021)	2	493	128687	0	0.91 [0.73, 1.14]	4.1E-01		0.39		Non-Significant	8
Soybean products (Fang, 2015)	6	6702	917650	0	0.88 [0.62, 1.26]	4.8E-01	0.35-2.23	0.5	0.965	Non-Significant	9
Spinach (Fang, 2015)	2	469	603453	0	1.25 [1.00, 1.56]	4.7E-02	0.73-2.14	0.05	0.270	Weak (IV)	9
Sweat confectionary (Fang, 2015)	3	1600	658075	0	1.24 [0.92, 1.67]	1.6E-01	0.64-2.4	0	0.973	Non-Significant	9
Tissue selenium (Cai, 2016)	5	1158	49790	1	0.79 [0.64, 0.98]	3.1E-02	0.46-1.35	0.65	0.273	Weak (IV)	8
Tofu (Fang, 2015)	6	2083	1254468	0	0.97 [0.78, 1.21]	7.9E-01	0.61-1.53	0.24	0.774	Non-Significant	9
Total carbohydrates (Fang, 2015)	4	2803	669965	1	1.17 [0.92, 1.49]	2.0E-01	0.51-2.7	0.34	0.439	Non-Significant	9
Total fat (Fang, 2015)	2	1251	927293	0	1.08 [0.80, 1.44]	6.2E-01	0.16-7.25	0	0.377	Non-Significant	9
Total soy food (Wang, 2021)	3	2011	116093	0	0.90 [0.69, 1.17]	4.2E-01	0.38-2.09	0.63	0.995	Non-Significant	8
Vegetable (Wang, 2014)	13	6323	2453435	0	0.96 [0.88, 1.06]	4.1E-01	0.77-1.19	0.21	0.062	Non-Significant	9

Vitamin A (Fang, 2015)	2	514	512051	1	1.06 [0.15, 7.31]	9.6E-01		0.92		Non-Significant	9
Vitamin B6 (Mocellin, 2017)	2	1262	1057585	0	1.02 [0.83, 1.25]	8.6E-01	0.27-3.8	0	0.529	Non-Significant	7
Vitamin C (Peiwei, 2015)	4	795	66095	1	0.77 [0.61, 0.97]	2.9E-02	0.46-1.29	0	0.823	Weak (IV)	6
Vitamin E (Kong, 2014)	3	1247	749853	1	0.81 [0.69, 0.95]	1.1E-02	0.28-2.31	0	0.765	Weak (IV)	7
Vodka (Fang, 2015)	2	444	481419	0	1.28 [0.86, 1.90]	2.2E-01		0		Non-Significant	9
Waist-to-hip ratio (Du, 2017)	4	1586	719858	0	1.33 [1.04, 1.70]	2.4E-02	0.71-2.47	0.36	0.378	Weak (IV)	9
Waist circumference (Du, 2017)	4	1707	844508	1	1.48 [1.24, 1.78]	2.3E-05	1.15-1.92	0	0.900	Suggestive (III)	9
White meat (Kim, 2019)	4	2387	1572442	0	0.99 [0.80, 1.22]	9.1E-01	0.54-1.82	0.56	0.569	Non-Significant	9
White vegetables (Fang, 2015)	2	544	80001	1	0.54 [0.32, 0.92]	2.2E-02		0		Weak (IV)-nominal**	9
Whole grains (Zhang, 2020)	2	1518	1004696	0	0.87 [0.72, 1.05]	1.5E-01	0.14-5.45	0.42	0.472	Non-Significant	7
Wine (Fang, 2015)	7	2645	1751844	0	1.07 [0.75, 1.51]	7.2E-01	0.39-2.92	0.6	0.018	Non-Significant	9
Yogurt (Fang, 2015)	2	709	566381	0	0.95 [0.67, 1.34]	7.7E-01	0.44-2.02	0	0.806	Non-Significant	9
**Gastric cardia cancer**											
Alcohol (Fang, 2015)	4	265	212633	1	1.04 [0.75, 1.44]	8.2E-01	0.29-3.72	0.55	0.290	Non-Significant	9
Beer (Fang, 2015)	2	96	47701	0	1.04 [0.59, 1.82]	8.9E-01		0		Non-Significant	9
Citrus fruit (Vingeliene, 2016)	3	631	324050	0	0.62 [0.39, 0.99]	4.4E-02	0-117.07	0.67	0.087	Weak (IV)	7
Fruit (Wang, 2014)	3	770	59182	0	0.89 [0.76, 1.04]	1.5E-01	0.32-2.46	0	0.781	Non-Significant	9
Liquor (Fang, 2015)	2	96	47701	1	1.38 [0.48, 3.96]	5.5E-01		0.66		Non-Significant	9
Obese vs normal (Chen, 2013)	7	3666	2073362	1	1.82 [1.32, 2.49]	2.1E-04	0.72-4.59	0.65	0.348	Suggestive (III)	9
Overweight vs normal (Chen, 2013)	6	1126	545443	0	1.21 [1.03, 1.42]	1.9E-02	0.97-1.51	0	0.376	Weak (IV)	9
Processed meat (Zhanwei Zhao, 2017)	3	547	379328	0	1.03 [0.70, 1.51]	8.9E-01	0.03-38.3	0.35	0.390	Non-Significant	9
Red meat (Zhanwei Zhao, 2017)	2	327	214430	0	1.07 [0.67, 1.70]	7.8E-01		0		Non-Significant	9
Tissue selenium (Cai, 2016)	2	704	5702	1	0.74 [0.50, 1.10]	1.3E-01		0.47		Non-Significant	8
Vegetable (Wang, 2014)	3	770	59182	0	1.12 [0.94, 1.35]	2.1E-01	0.34-3.65	0	0.235	Non-Significant	9
Waist-to-hip ratio (Du, 2017)	2	370	203437	0	1.41 [0.79, 2.51]	2.4E-01		0.64		Non-Significant	9
Waist circumference (Du, 2017)	2	370	203437	1	1.94 [1.30, 2.91]	1.3E-03		0		Weak (IV)-nominal**	9
White meat (Kim, 2019)	2	872	329927	0	1.09 [0.88, 1.35]	4.5E-01		0		Non-Significant	9
Wine (Fang, 2015)	2	96	47701	0	1.55 [0.57, 4.25]	3.9E-01		0.33		Non-Significant	9
**Gastric non-cardia cancer**											
Alcohol (Fang, 2015)	4	529	425267	0	1.07 [0.64, 1.78]	8.0E-01	0.11-10.06	0.8	0.506	Non-Significant	9
Beer (Fang, 2015)	2	192	95402	0	1.43 [0.91, 2.24]	1.2E-01		0		Non-Significant	9
Citrus fruit (Vingeliene, 2016)	5	1601	780410	0	1.01 [0.79, 1.28]	9.6E-01	0.49-2.08	0.47	0.279	Non-Significant	7
Fruit (Wang, 2014)	3	1541	118364	0	0.85 [0.70, 1.03]	1.0E-01	0.24-3	0	0.062	Non-Significant	9
Liquor (Fang, 2015)	2	192	95402	0	0.52 [0.30, 0.88]	1.6E-02		0.01		Weak (IV)-nominal**	9
Obese vs normal (Chen, 2013)	8	8391	3774380	0	1.00 [0.87, 1.15]	1.0E+00	0.74-1.35	0.29	0.992	Non-Significant	9
Overweight vs normal (Chen, 2013)	7	7647	1531354	1	0.93 [0.82, 1.05]	2.6E-01	0.67-1.28	0.54	0.803	Non-Significant	9
Processed meat (Zhanwei Zhao, 2017)	3	1093	758657	0	1.27 [0.94, 1.71]	1.2E-01	0.07-21.76	0.38	0.291	Non-Significant	9
Red meat (Zhanwei Zhao, 2017)	2	655	428861	0	1.31 [0.93, 1.85]	1.2E-01		0.14		Non-Significant	9
Vegetable (Wang, 2014)	3	1541	118364	0	0.94 [0.74, 1.20]	6.4E-01	0.2-4.42	0	0.787	Non-Significant	9
Waist-to-hip ratio (Du, 2017)	3	838	448168	0	1.42 [0.90, 2.23]	1.3E-01	0.01-139.7	0.49	0.393	Non-Significant	9
Waist circumference (Du, 2017)	3	838	448168	0	1.24 [0.88, 1.75]	2.2E-01	0.13-11.47	0	0.678	Non-Significant	9
White meat (Kim, 2019)	2	1011	659121	0	0.85 [0.69, 1.05]	1.2E-01		0		Non-Significant	9
Wine (Fang, 2015)	2	192	95402	0	1.71 [0.37, 7.81]	4.9E-01		0.75		Non-Significant	9

*The contrast for all these associations was reported as the highest versus lowest categories of exposure; for several exposures associations both case-control and prospective primary data were available but only analyses based on prospective primary studies were subject to the classification process.

**In case only two primary studies were available and random-effects MA P-value was <0.05, then evidence was classified as weak since it would not be possible to evaluate using the full set of validity criteria.

ID: Exposure; K_studies: Total primary studies; Total_cases: Total cases; Participants: Total participants; statSig0: Largest primary study with significant (P<0.05) estimate; Effect*: Random Effects Meta-analysis, Relative risk & 95% confidence Interval; Pval: P-value; PI: Prediction Intervals; I^2: Inconsistency index; Pegger: Egger's regression P-value; ES_p: Excess significance P-value; class: Classification of evidence; TP: AMSTAR points

Table S4. Associations between diet and gastric cancer and subtypes from the sensitivity analysis including both prospective and case-control primary studies.

ID	k_studies	Total_cases	Participants	statSig0	Effect*	Pval	PI	I2	Pegger	class	TP
**Gastric cancer**											
a-carotene (Fang, 2015)	2	1345	56209	0	0.78 [0.33, 1.85]	5.8E-01		0.85		Non-Significant	9
Alcohol (Deng, 2021)	81	167033	25283325	1	1.20 [1.12, 1.27]	2.7E-08	0.75-1.9	0.86	0.000	Suggestive (III)	5
Alcohol ≥42 grams/day (Deng, 2021)	29	24923	2797576	1	1.30 [1.17, 1.44]	1.0E-06	0.84-2.01	0.78	0.428	Suggestive (III)	5
Allium vegetables (Turati, 2015)	14	6210	707870	0	0.79 [0.68, 0.91]	1.3E-03	0.49-1.26	0.56	0.465	Weak (IV)	5
Animal fat (Han, 2015)	5	2808	6662	0	1.10 [0.90, 1.33]	3.5E-01	0.66-1.82	0.42	0.991	Non-Significant	9
Anthocyanin (Yang, 2019)	5	3133	950996	0	0.89 [0.77, 1.02]	9.3E-02	0.74-1.07	0	0.491	Non-Significant	7
Antioxidant Supplements, b-carotene, vitamins A, C, E, and Se (Bjelakovic, 2008)	12	714	157300	0	1.14 [0.98, 1.33]	1.0E-01	0.95-1.36	0	0.660	Non-Significant	11
Apples and/or pears (Fang, 2015)	3	531	212839	0	1.02 [0.95, 1.10]	5.4E-01	0.79-1.33	0.66	0.704	Non-Significant	9
b-carotene (Peiwei, 2015)	20	6281	128411	1	0.59 [0.50, 0.70]	1.1E-09	0.31-1.12	0.68	0.978	Suggestive (III)	6
b-cryptoxanthin (Fang, 2015)	2	1345	56209	0	1.09 [0.78, 1.53]	6.2E-01		0		Non-Significant	9
Bacon or side pork (Fang, 2015)	2	1473	159428	0	1.51 [1.03, 2.22]	3.5E-02		0		Weak (IV)-nominal**	9
Beans (Fang, 2015)	2	519	531436	0	0.82 [0.62, 1.10]	1.9E-01	0.13-5.31	0	0.840	Non-Significant	9
Beer (Fang, 2015)	9	2766	1782148	0	1.17 [0.99, 1.37]	5.9E-02	0.83-1.64	0.24	0.291	Non-Significant	9
Black Tea (Fang, 2015)	3	1113	606374	0	1.20 [0.81, 1.77]	3.5E-01	0.42-3.46	0.39	0.822	Non-Significant	9
Blood retinol (Wu, 2015)	5	1561	33253	0	0.87 [0.71, 1.08]	2.0E-01	0.47-1.62	0.47	0.956	Non-Significant	8
Brassica vegetables (Fang, 2015)	2	328	91987	0	0.82 [0.59, 1.14]	2.4E-01	0.03-20.85	0.41	0.208	Non-Significant	9
Bread (Fang, 2015)	4	1912	676319	0	1.15 [0.90, 1.48]	2.5E-01	0.6-2.21	0.29	0.089	Non-Significant	9
Cabbage (Qi, 2013)	7	2313	77250	1	0.68 [0.58, 0.80]	1.6E-06	0.56-0.84	0	0.125	Suggestive (III)	8
Capsaicin (Pabalan, 2014)	9	2441	5672	1	0.55 [0.37, 0.82]	2.8E-03	0.14-2.24	0.9	0.785	Weak (IV)	7
Carotenoid (Zhou, 2016)	27	4196	16147	1	0.62 [0.52, 0.74]	5.2E-08	0.3-1.3	0.62	0.838	Suggestive (III)	7
Cheese (Fang, 2015)	2	709	566381	0	1.02 [0.66, 1.58]	9.4E-01	0.39-2.67	0	0.422	Non-Significant	9
Chicken (Fang, 2015)	3	862	641323	0	1.12 [0.86, 1.46]	4.0E-01	0.73-1.72	0	0.333	Non-Significant	9
Chili (Du, 2020)	13	3095	7856	1	1.96 [1.59, 2.42]	2.8E-10	0.94-4.1	0.75	0.500	Suggestive (III)	9
Chili pepper (Luo, 2021)	16	3317	11654	1	1.96 [1.34, 2.86]	4.7E-04	0.44-8.71	0.81	0.594	Suggestive (III)	7
Chinese chives (Turati, 2015)	4	1595	3838	1	0.44 [0.26, 0.73]	1.6E-03	0.05-3.72	0.69	0.290	Weak (IV)	5
Cholesterol (Miao, 2021)	14	6692	26433	1	1.35 [1.13, 1.62]	1.0E-03	0.74-2.49	0.7	0.828	Weak (IV)	10
Citrus fruit (Vingeliene, 2016)	8	5446	2219490	0	0.94 [0.83, 1.05]	2.6E-01	0.69-1.27	0.47	0.387	Non-Significant	7
Coffee (Deng, 2016)	14	3484	1324559	0	1.16 [0.99, 1.35]	6.6E-02	0.8-1.68	0.27	0.290	Non-Significant	8

Cruciferous vegetables (Qi, 2013)	21	7113	831555	1	0.81 [0.75, 0.88]	1.2E-06	0.68-0.97	0.12	0.668	Suggestive (III)	8
Dairy (Guo, 2015)	13	3967	606302	1	1.09 [0.83, 1.43]	5.5E-01	0.36-3.25	0.85	0.583	Non-Significant	7
Dietary fiber (Zhang, 2013)	21	6950	580658	0	0.58 [0.49, 0.67]	1.3E-12	0.31-1.08	0.62	0.931	Suggestive (III)	9
Dietary Inflammatory Index (Moazzen, 2020)	4	1546	520012	0	0.85 [0.49, 1.46]	5.6E-01	0.07-10.33	0.9	0.271	Non-Significant	9
Dietary iron (Fonseca-Nunes, 2014)	4	817	30733	0	0.82 [0.45, 1.48]	5.0E-01	0.07-9.72	0.66	0.560	Non-Significant	5
Dried or salted fish (Fang, 2015)	3	4221	1261988	1	1.27 [1.05, 1.52]	1.2E-02	0.94-1.71	0	0.037	Weak (IV)	9
Egg (Tse, 2014)	18	5919	162798	0	1.13 [0.90, 1.42]	2.9E-01	0.44-2.92	0.8	0.158	Non-Significant	6
Fish (Fang, 2015)	14	7117	1835050	0	1.11 [0.99, 1.23]	6.9E-02	0.98-1.24	0	0.772	Non-Significant	9
Flavan-3-ols (Grosso, 2017)	5	2519	957320	0	0.88 [0.68, 1.13]	3.1E-01	0.45-1.71	0.45	0.209	Non-Significant	8
Flavanones (Grosso, 2017)	5	2519	957320	0	0.97 [0.82, 1.15]	7.2E-01	0.78-1.21	0	0.609	Non-Significant	8
Flavones (Grosso, 2017)	4	1836	480008	0	0.82 [0.64, 1.04]	1.0E-01	0.44-1.51	0.4	0.135	Non-Significant	8
Flavonols (Grosso, 2017)	4	1836	480008	0	0.85 [0.64, 1.14]	2.8E-01	0.37-1.95	0.57	0.623	Non-Significant	8
Folate (Tio, 2014)	16	4414	209689	1	0.94 [0.78, 1.13]	5.1E-01	0.49-1.78	0.55	0.315	Non-Significant	7
Fruit (Fang, 2015)	24	10776	3805553	0	0.93 [0.89, 0.98]	4.1E-03	0.86-1.01	0.04	0.186	Weak (IV)	9
Fruiting vegetables (Fang, 2015)	3	1422	898691	0	0.94 [0.76, 1.15]	5.5E-01	0.25-3.58	0	0.266	Non-Significant	9
Garlic (Turati, 2015)	13	3745	10438	0	0.61 [0.48, 0.77]	3.6E-05	0.3-1.23	0.54	0.863	Suggestive (III)	5
Glycemic Index (Ye, 2016)	6	50857	517764	0	1.17 [0.80, 1.69]	4.2E-01	0.34-3.98	0.83	0.877	Non-Significant	7
Glycemic Load (Ye, 2016)	6	50857	517764	0	1.10 [0.85, 1.42]	4.6E-01	0.56-2.15	0.45	0.408	Non-Significant	7
Green-yellow vegetables (Fang, 2015)	3	2024	130837	0	1.05 [0.90, 1.21]	5.6E-01	0.4-2.71	0	0.796	Non-Significant	9
Green and yellow vegetable (Fang, 2015)	2	467	128002	1	1.16 [0.14, 9.65]	8.9E-01		0.91		Non-Significant	9
Green leafy vegetables (Fang, 2015)	2	1125	50334	1	0.75 [0.54, 1.05]	9.4E-02		0.12		Non-Significant	9
Green tea (Huang, 2017)	13	6627	376943	0	0.89 [0.76, 1.03]	1.2E-01	0.58-1.36	0.49	0.124	Non-Significant	9
Green vegetables (Fang, 2015)	2	475	84397	0	0.72 [0.47, 1.10]	1.3E-01		0		Non-Significant	9
Healthy lifestyle score (Zhang, 2020)	6	2974	1181751	1	0.60 [0.48, 0.74]	4.8E-06	0.3-1.17	0.62	0.370	Suggestive (III)	9
Heme iron (Fonseca-Nunes, 2014)	3	1130	785178	1	1.16 [0.71, 1.89]	5.6E-01	0.13-10.09	0.79	0.981	Non-Significant	5
Highly salted food (Fang, 2015)	2	1044	117942	0	2.12 [0.59, 7.62]	2.5E-01	0-15575516.55	0.88	0.714	Non-Significant	9
Isoflavones (Lu, 2017)	2	1927	115673	0	0.90 [0.73, 1.10]	2.9E-01	0.44-1.83	0.4	0.293	Non-Significant	6
Isoflavones (Wang, 2021)	8	4864	588718	0	0.92 [0.79, 1.07]	3.0E-01	0.6-1.41	0.51	0.909	Non-Significant	8
Kaempferol (Grosso, 2017)	2	428	10573	1	0.73 [0.31, 1.71]	4.7E-01		0.72		Non-Significant	8

Leafy vegetables (Fang, 2015)	2	1179	869558	0	1.03 [0.81, 1.30]	8.2E-01		0		Non-Significant	9
Legumes and pulses (Fang, 2015)	4	2652	1194100	0	0.88 [0.74, 1.05]	1.5E-01	0.67-1.17	0	0.278	Non-Significant	9
Lignans (Grosso, 2017)	3	1483	560233	0	0.90 [0.73, 1.11]	3.2E-01	0.64-1.27	0	0.618	Non-Significant	8
Liquor (Fang, 2015)	7	2322	1300729	0	1.11 [0.85, 1.44]	4.5E-01	0.54-2.26	0.51	0.265	Non-Significant	9
Liver (Fang, 2015)	3	991	687233	1	1.34 [0.63, 2.83]	4.5E-01	0.16-11.09	0.41	0.858	Non-Significant	9
Lutein (Zhou, 2016)	5	1068	3992	0	0.89 [0.60, 1.32]	5.7E-01	0.29-2.77	0.46	0.542	Non-Significant	7
Lutein and zeaxanthin (Fang, 2015)	2	1345	56209	0	0.99 [0.57, 1.72]	9.7E-01		0.63		Non-Significant	9
Lycopene (Fang, 2015)	3	1467	84672	0	0.88 [0.67, 1.16]	3.8E-01	0.49-1.61	0	0.927	Non-Significant	9
Margarine (Fang, 2015)	2	612	642303	0	0.75 [0.56, 1.02]	6.3E-02	0.11-5.21	0	0.895	Non-Significant	9
Meat (Fang, 2015)	11	5393	1611578	0	1.00 [0.89, 1.11]	9.5E-01	0.79-1.26	0.24	0.960	Non-Significant	9
Meat products (Fang, 2015)	8	3030	758084	0	1.00 [0.84, 1.20]	9.7E-01	0.64-1.58	0.4	0.908	Non-Significant	9
Mediterranean Diet Score (Schwingshackl, 2017)	4	2625	985488	1	0.71 [0.60, 0.86]	2.9E-04	0.41-1.26	0.57	0.531	Suggestive (III)	7
Milk (Fang, 2015)	5	2133	1300378	0	1.06 [0.87, 1.28]	5.8E-01	0.71-1.58	0.24	0.554	Non-Significant	9
Miso soup (Fang, 2015)	10	8501	2216989	0	1.12 [0.98, 1.28]	1.0E-01	0.82-1.52	0.31	0.832	Non-Significant	9
Mono-unsaturated fatty acids (Han, 2015)	11	4224	508551	0	1.00 [0.79, 1.25]	9.7E-01	0.48-2.08	0.63	0.670	Non-Significant	9
Mushroom (Fang, 2015)	3	976	579144	0	0.97 [0.74, 1.28]	8.4E-01	0.62-1.52	0	0.407	Non-Significant	9
Myricetin (Grosso, 2017)	2	428	10573	0	1.13 [0.75, 1.71]	5.5E-01		0		Non-Significant	8
NDMA (Song, 2015)	8	2738	351866	1	1.34 [1.02, 1.76]	3.2E-02	0.55-3.3	0.76	0.692	Weak (IV)	9
Nitrates (Song, 2015)	15	5036	652559	1	0.80 [0.69, 0.93]	3.5E-03	0.5-1.28	0.46	0.047	Weak (IV)	9
Nitrites (Song, 2015)	14	5758	641816	0	1.31 [1.13, 1.52]	2.9E-04	0.81-2.13	0.47	0.542	Suggestive (III)	9
Non-citrus fruit (Fang, 2015)	2	1273	39445	0	0.98 [0.95, 1.02]	2.8E-01		0		Non-Significant	9
Nut consumption (Zhang, 2020)	5	2868	691035	1	0.89 [0.69, 1.13]	3.4E-01	0.41-1.91	0.76	0.548	Non-Significant	6
Obese vs normal (Chen, 2013)	20	35023	10281810	0	1.15 [1.02, 1.29]	1.9E-02	0.75-1.76	0.65	0.165	Weak (IV)	9
Olive oil (Pelucchi, 2011)	3	1142	2862	1	0.76 [0.63, 0.92]	4.2E-03	0.23-2.56	0	0.001	Weak (IV)	2
Onion & Garlic (Fang, 2015)	2	1179	869558	0	0.92 [0.74, 1.14]	4.3E-01		0		Non-Significant	9
Onion (Turati, 2015)	14	4557	11416	1	0.55 [0.41, 0.73]	3.2E-05	0.2-1.47	0.76	0.053	Suggestive (III)	5
Overweight vs normal (Chen, 2013)	15	31535	6182768	0	1.01 [0.95, 1.07]	8.4E-01	0.86-1.18	0.43	0.408	Non-Significant	9
Pickled vegetables (Fang, 2015)	7	6803	970667	0	1.36 [1.03, 1.79]	3.1E-02	0.57-3.22	0.71	0.611	Weak (IV)	9
Pickles (Fang, 2015)	7	2832	1802230	0	1.09 [0.99, 1.20]	7.6E-02	0.97-1.23	0	0.783	Non-Significant	9
Plasma vitamin C (Lam, 2013)	3	902	3065	1	0.73 [0.58, 0.91]	5.7E-03	0.17-3.1	0	0.922	Weak (IV)	3
Poly-unsaturated fatty acids (Han, 2015)	12	4831	509845	0	0.77 [0.65, 0.92]	3.4E-03	0.44-1.36	0.56	0.063	Weak (IV)	9
Potato (Fang, 2015)	4	1382	705477	0	0.96 [0.92, 1.00]	5.6E-02	0.91-1.02	0	0.970	Non-Significant	9

Poultry (Fang, 2015)	2	1917	604978	1	0.96 [0.39, 2.38]	9.3E-01		0.84		Non-Significant	9
Proanthocyanidins (Grosso, 2017)	2	913	478089	0	0.60 [0.36, 1.01]	5.4E-02	0-243.67	0.72	0.250	Non-Significant	8
Processed meat (Zhanwei Zhao, 2017)	33	10434	1330108	1	1.61 [1.41, 1.84]	1.9E-12	0.9-2.87	0.58	0.006	Suggestive (III)	9
Quercetin (Grosso, 2017)	3	933	12194	1	0.66 [0.51, 0.85]	1.1E-03	0.38-1.14	0	0.150	Weak (IV)	8
Raw vegetables (Fang, 2015)	3	2450	222378	0	0.96 [0.77, 1.21]	7.5E-01	0.67-1.39	0	0.012	Non-Significant	9
Red meat (Zhanwei Zhao, 2017)	24	9851	808298	0	1.55 [1.31, 1.84]	3.5E-07	0.78-3.11	0.68	0.020	Suggestive (III)	9
Refined grains (Wang, 2020)	16	5221	21409	1	1.38 [1.22, 1.56]	3.3E-07	0.92-2.07	0.57	0.456	Suggestive (III)	8
Refined non-rice grain (Wang, 2020)	9	4303	13447	1	1.28 [1.11, 1.49]	9.5E-04	0.82-2.02	0.67	0.765	Suggestive (III)	8
Retinol (Wu, 2015)	19	5706	285921	0	0.90 [0.79, 1.03]	1.2E-01	0.6-1.35	0.45	0.123	Non-Significant	8
Rice (Fang, 2015)	3	2721	1043328	0	1.08 [0.89, 1.31]	4.6E-01	0.7-1.65	0	0.850	Non-Significant	9
Root vegetables (Fang, 2015)	2	775	332289	0	0.66 [0.30, 1.49]	3.2E-01		0.89		Non-Significant	9
Salt (Fang, 2015)	7	6553	2301836	1	1.14 [1.01, 1.28]	3.5E-02	0.88-1.47	0.24	0.310	Weak (IV)	9
Salted fish (Fang, 2015)	6	5022	1039934	1	1.56 [1.30, 1.87]	1.4E-06	1.24-1.96	0.01	0.572	Suggestive (III)	9
Salted fish roe (Fang, 2015)	2	3891	740537	1	1.68 [1.37, 2.06]	7.9E-07	0.44-6.38	0	0.682	Weak (IV)	9
Salted food (Fang, 2015)	6	3128	1673808	0	1.30 [1.04, 1.63]	2.1E-02	0.98-1.73	0	0.042	Weak (IV)	9
Saturated fatty acids (Han, 2015)	14	5046	510641	0	1.34 [1.12, 1.60]	1.4E-03	0.71-2.52	0.6	0.092	Weak (IV)	9
Sausage (Fang, 2015)	2	1600	560027	0	1.49 [1.10, 2.02]	9.6E-03	0.21-10.7	0	0.278	Weak (IV)	9
Seaweed (Fang, 2015)	5	2197	1192289	0	1.02 [0.83, 1.25]	8.7E-01	0.78-1.33	0	0.461	Non-Significant	9
Serum lycopene (Yang, 2013)	3	946	2672	0	0.78 [0.56, 1.09]	1.5E-01	0.05-12.35	0.2	0.175	Non-Significant	5
Serum Vitamin D (Khayatzadeh, 2015)	2	512	50407	0	1.02 [0.76, 1.37]	8.9E-01	0.12-8.65	0.07	0.424	Non-Significant	8
Serum/plasma a-carotene (Zhou, 2016)	3	946	20346	0	0.79 [0.47, 1.31]	3.5E-01	0-147.89	0.53	0.688	Non-Significant	7
Serum/plasma b-carotene (Zhou, 2016)	7	1952	93512	0	0.72 [0.49, 1.06]	9.4E-02	0.22-2.38	0.69	0.679	Non-Significant	7
Serum/plasma carotenoids (Zhou, 2016)	17	1952	93512	0	0.80 [0.66, 0.96]	1.8E-02	0.43-1.49	0.53	0.065	Weak (IV)	7
Serum/plasma lutein (Zhou, 2016)	4	1428	21881	0	0.97 [0.76, 1.26]	8.4E-01	0.44-2.15	0.25	0.310	Non-Significant	7
Serum/plasma lycopene (Zhou, 2016)	3	946	20346	0	0.78 [0.56, 1.09]	1.5E-01	0.05-12.35	0.2	0.175	Non-Significant	7
Soy bean (Wang, 2021)	3	870	129818	0	0.84 [0.68, 1.05]	1.4E-01	0.09-7.98	0.48	0.663	Non-Significant	8
Soy milk (Wang, 2021)	3	1006	130090	0	0.69 [0.50, 0.95]	2.5E-02	0.02-20.38	0.56	0.256	Weak (IV)	8
Soy sprouts (Wang, 2021)	2	870	129818	0	0.97 [0.79, 1.19]	7.9E-01		0		Non-Significant	8

Soybean paste (Wang, 2021)	5	1735	51642	1	1.39 [0.85, 2.30]	1.9E-01	0.27-7.09	0.8	0.932	Non-Significant	8
Soybean products (Fang, 2015)	6	6702	917650	0	0.88 [0.62, 1.26]	4.8E-01	0.35-2.23	0.5	0.965	Non-Significant	9
Spinach (Fang, 2015)	2	469	603453	0	1.25 [1.00, 1.56]	4.7E-02	0.73-2.14	0.05	0.270	Weak (IV)	9
Sweat confectionary (Fang, 2015)	3	1600	658075	0	1.24 [0.92, 1.67]	1.6E-01	0.64-2.4	0	0.973	Non-Significant	9
Tissue selenium (Cai, 2016)	5	1158	49790	1	0.79 [0.64, 0.98]	3.1E-02	0.46-1.35	0.65	0.273	Weak (IV)	8
Tofu (Wang, 2021)	8	3246	223800	0	0.72 [0.55, 0.93]	1.1E-02	0.34-1.51	0.68	0.019	Weak (IV)	8
Tomato (Yang, 2013)	9	2596	10089	0	0.73 [0.61, 0.88]	1.1E-03	0.43-1.23	0.46	0.166	Weak (IV)	5
Total carbohydrates (Ye, 2016)	22	5905	83612	1	1.17 [0.91, 1.50]	2.1E-01	0.4-3.4	0.79	0.784	Non-Significant	7
Total fat (Han, 2015)	21	8430	523262	1	1.18 [1.00, 1.38]	4.6E-02	0.58-2.39	0.68	0.397	Weak (IV)	9
Total flavonoids (Grosso, 2017)	3	1606	479231	0	0.89 [0.66, 1.20]	4.6E-01	0.39-2.06	0.52	0.401	Non-Significant	8
Total Reactive Antioxidant Potential (Parohan, 2019)	2	735	2308	1	0.62 [0.48, 0.81]	3.0E-04		0		Weak (IV)-nominal**	9
Total soy food (Wang, 2021)	13	5354	125219	0	0.62 [0.49, 0.79]	1.1E-04	0.25-1.56	0.8	0.044	Suggestive (III)	8
Vegetable (Wang, 2014)	13	6323	2453435	0	0.96 [0.88, 1.06]	4.1E-01	0.77-1.19	0.21	0.062	Non-Significant	9
Vegetable fat (Han, 2015)	4	1684	4087	1	0.55 [0.41, 0.74]	7.8E-05	0.18-1.66	0.49	0.960	Suggestive (III)	9
Vitamin A (Wu, 2015)	15	2796	91526	1	0.66 [0.52, 0.84]	7.0E-04	0.3-1.48	0.65	0.350	Suggestive (III)	8
Vitamin B1 (Kong, 2014)	2	228	492	0	0.82 [0.50, 1.35]	4.4E-01		0.51		Non-Significant	7
Vitamin B2 (Kong, 2014)	2	228	492	0	0.72 [0.35, 1.47]	3.6E-01		0.78		Non-Significant	7
Vitamin B6 (Mocellin, 2017)	8	2228	1060481	0	0.75 [0.63, 0.89]	1.1E-03	0.47-1.21	0.46	0.849	Weak (IV)	7
Vitamin C (Peiwei, 2015)	32	9507	97358	1	0.58 [0.51, 0.65]	5.5E-19	0.36-0.91	0.46	0.658	Highly Suggestive (II)	6
Vitamin D (Khayatzadeh, 2015)	4	1652	5038	0	1.09 [0.93, 1.28]	2.6E-01	0.79-1.52	0.13	0.346	Non-Significant	8
Vitamin E (Peiwei, 2015)	24	8130	577359	1	0.65 [0.57, 0.74]	3.0E-10	0.41-1.03	0.48	0.063	Suggestive (III)	6
Vodka (Fang, 2015)	2	444	481419	0	1.28 [0.86, 1.90]	2.2E-01		0		Non-Significant	9
Waist-to-hip ratio (Du, 2017)	4	1586	719858	0	1.33 [1.04, 1.70]	2.4E-02	0.71-2.47	0.36	0.378	Weak (IV)	9
Waist circumference (Du, 2017)	4	1707	844508	1	1.48 [1.24, 1.78]	2.3E-05	1.15-1.92	0	0.900	Suggestive (III)	9
White meat (Kim, 2019)	15	8005	1590669	0	0.86 [0.75, 0.98]	2.4E-02	0.57-1.28	0.39	0.112	Weak (IV)	9
White vegetables (Fang, 2015)	2	544	80001	1	0.54 [0.32, 0.92]	2.2E-02		0		Weak (IV)-nominal**	9
Whole grains (Zhang, 2020)	10	8274	1021955	0	0.64 [0.53, 0.79]	2.1E-05	0.33-1.26	0.78	0.009	Suggestive (III)	7
Wine (Fang, 2015)	7	2645	1751844	0	1.07 [0.75, 1.51]	7.2E-01	0.39-2.92	0.6	0.018	Non-Significant	9
Yogurt (Fang, 2015)	2	709	566381	0	0.95 [0.67, 1.34]	7.7E-01	0.44-2.02	0	0.806	Non-Significant	9
Zinc (Li, 2014)	7	1609	4128	1	0.91 [0.64, 1.29]	5.8E-01	0.3-2.74	0.78	0.575	Non-Significant	6
**Gastric cardia cancer**											
Alcohol (Fang, 2015)	4	265	212633	1	1.04 [0.75, 1.44]	8.2e-01	0.29-3.72	0.55	0.290	Non-Significant	9
Anthocyanin (Yang, 2019)	2	873	157430	0	0.90 [0.62, 1.31]	5.7e-01		0.55		Non-Significant	7
Beer (Fang, 2015)	2	96	47701	0	1.04 [0.59, 1.82]	8.9e-01		0		Non-Significant	9
Citrus fruit (Vingeliene, 2016)	3	631	324050	0	0.62 [0.39, 0.99]	4.4e-02	0-117.07	0.67	0.087	Weak (IV)	7

Dietary fiber (Zhang, 2013)	2	512	1177	1	0.49 [0.37, 0.66]	1.7e-06		0.16		Weak (IV)-nominal**	9
Fruit (Wang, 2014)	3	770	59182	0	0.89 [0.76, 1.04]	1.5e-01	0.32-2.46	0	0.781	Non-Significant	9
Liquor (Fang, 2015)	2	96	47701	1	1.38 [0.48, 3.96]	5.5e-01		0.66		Non-Significant	9
Mono-unsaturated fatty acids (Han, 2015)	2	526	165955	0	1.15 [0.88, 1.52]	3.1e-01		0		Non-Significant	9
Obese vs normal (Chen, 2013)	7	3666	2073362	1	1.82 [1.32, 2.49]	2.1e-04	0.72-4.59	0.65	0.348	Suggestive (III)	9
Overweight vs normal (Chen, 2013)	6	1126	545443	0	1.21 [1.03, 1.42]	1.9e-02	0.97-1.51	0	0.376	Weak (IV)	9
Poly-unsaturated fatty acids (Han, 2015)	3	728	166386	0	0.98 [0.79, 1.20]	8.2e-01	0.25-3.74	0	0.446	Non-Significant	9
Processed meat (Zhanwei Zhao, 2017)	5	844	380196	1	1.06 [0.85, 1.33]	5.9e-01	0.58-1.95	0.39	0.759	Non-Significant	9
Red meat (Zhanwei Zhao, 2017)	4	625	215298	1	1.42 [1.14, 1.77]	1.6e-03	0.88-2.3	0	0.271	Weak (IV)	9
Saturated fatty acids (Han, 2015)	3	728	166386	0	1.20 [0.95, 1.52]	1.2e-01	0.27-5.45	0	0.077	Non-Significant	9
Tissue selenium (Cai, 2016)	2	704	5702	1	0.74 [0.50, 1.10]	1.3e-01		0.47		Non-Significant	8
Tomato (Yang, 2013)	2	365	2099	0	0.83 [0.57, 1.21]	3.3e-01		0.33		Non-Significant	5
Total fat (Han, 2015)	5	1082	167656	0	1.23 [1.01, 1.50]	4.2e-02	0.89-1.7	0	0.778	Weak (IV)	9
Vegetable (Wang, 2014)	3	770	59182	0	1.12 [0.94, 1.35]	2.1e-01	0.34-3.65	0	0.235	Non-Significant	9
Vitamin B6 (Mocellin, 2017)	2	572	492527	1	0.77 [0.55, 1.08]	1.3e-01		0.56		Non-Significant	7
Waist-to-hip ratio (Du, 2017)	2	370	203437	0	1.41 [0.79, 2.51]	2.4e-01		0.64		Non-Significant	9
Waist circumference (Du, 2017)	2	370	203437	1	1.94 [1.30, 2.91]	1.3e-03		0		Weak (IV)-nominal**	9
White meat (Kim, 2019)	3	1177	330737	0	1.08 [0.88, 1.32]	4.8e-01	0.28-4.09	0	0.734	Non-Significant	9
Wine (Fang, 2015)	2	96	47701	0	1.55 [0.57, 4.25]	3.9e-01		0.33		Non-Significant	9
**Gastric non-cardia cancer**											
Alcohol (Ferro, 2018)	3	1253	2741	0	1.05 [0.85, 1.29]	6.5e-01	0.65-1.7	0.21	0.634	Non-Significant	7
Anthocyanin (Yang, 2019)	2	1013	314126	0	0.85 [0.65, 1.11]	2.2e-01		0.25		Non-Significant	7
Beer (Fang, 2015)	2	192	95402	0	1.43 [0.91, 2.24]	1.2e-01		0		Non-Significant	9
Citrus fruit (Vingeliene, 2016)	5	1601	780410	0	1.01 [0.79, 1.28]	9.6e-01	0.49-2.08	0.47	0.279	Non-Significant	7
Dietary fiber (Zhang, 2013)	2	718	2048	1	0.51 [0.28, 0.92]	2.4e-02		0.84		Weak (IV)-nominal**	9
Fruit (Wang, 2014)	3	1541	118364	0	0.85 [0.70, 1.03]	1.0e-01	0.24-3	0	0.062	Non-Significant	9
Liquor (Fang, 2015)	2	192	95402	0	0.52 [0.30, 0.88]	1.6e-02		0.01		Weak (IV)-nominal**	9
Mono-unsaturated fatty acids (Han, 2015)	2	1052	331909	0	1.15 [0.68, 1.93]	6.0e-01		0.72		Non-Significant	9
Obese vs normal (Chen, 2013)	8	8391	3774380	0	1.00 [0.87, 1.15]	1.0e+00	0.74-1.35	0.29	0.992	Non-Significant	9
Overweight vs normal (Chen, 2013)	7	7647	1531354	1	0.93 [0.82, 1.05]	2.6e-01	0.67-1.28	0.54	0.803	Non-Significant	9

Poly-unsaturated fatty acids (Han, 2015)	3	1457	332772	0	0.85 [0.67, 1.09]	2.0e-01	0.08-8.7	0.39	0.750	Non-Significant	9
Processed meat (Zhanwei Zhao, 2017)	6	1813	760966	1	1.38 [1.17, 1.62]	1.1e-04	1.03-1.84	0.09	0.415	Suggestive (III)	9
Red meat (Zhanwei Zhao, 2017)	5	1374	431170	1	1.52 [1.24, 1.87]	5.8e-05	1.09-2.13	0	0.513	Suggestive (III)	9
Saturated fatty acids (Han, 2015)	3	1457	332772	0	1.39 [1.13, 1.72]	2.0e-03	0.35-5.48	0	0.114	Weak (IV)	9
Tomato (Yang, 2013)	2	731	4197	0	0.93 [0.66, 1.30]	6.6e-01		0.33		Non-Significant	5
Total fat (Han, 2015)	6	1840	338351	1	1.31 [1.14, 1.51]	1.5e-04	1.08-1.61	0	0.688	Suggestive (III)	9
Vegetable (Wang, 2014)	3	1541	118364	0	0.94 [0.74, 1.20]	6.4e-01	0.2-4.42	0	0.787	Non-Significant	9
Vitamin B6 (Mocellin, 2017)	2	539	492488	0	0.82 [0.43, 1.54]	5.3e-01		0.9		Non-Significant	7
Waist-to-hip ratio (Du, 2017)	3	838	448168	0	1.42 [0.90, 2.23]	1.3e-01	0.01-139.7	0.49	0.393	Non-Significant	9
Waist circumference (Du, 2017)	3	838	448168	0	1.24 [0.88, 1.75]	2.2e-01	0.13-11.47	0	0.678	Non-Significant	9
White meat (Kim, 2019)	3	1621	660740	0	0.83 [0.68, 1.02]	7.8e-02	0.22-3.09	0	0.393	Non-Significant	9
Wine (Fang, 2015)	2	192	95402	0	1.71 [0.37, 7.81]	4.9e-01		0.75		Non-Significant	9

*The contrast for all these associations was reported as the highest versus lowest categories of exposure; for several exposures associations both case-control and prospective primary data were available but only analyses based on prospective primary studies were subject to the classification process

ID: Exposure; K_studies: Total primary studies; Total_cases: Total cases; Participants: Total participants; statSig0: Largest primary study with significant (P<0.05) estimate; Effect: Random Effects Meta-analysis, Relative risk & 95% confidence Interval; Pval: P-value; PI: Prediction Intervals; I^2: Inconsistency index; Pegger: Egger's regression P-value; ES_p: Excess significance P-value; class: Classification of evidence; TP: AMSTAR points

Supplementary Table S5. AMSTAR classification.

Study	1.Was an 'apriori' design provided	2.Was there duplicate study selection and data extraction	3.Was a comprehensive literature search performed	4.Was the status of publication (i.e. grey literature) used as an inclusion criterion	5.Was a list of studies (included and excluded) provided	6.Were the characteristics of the included studies provided	7.Was the scientific quality of the included studies assessed and documented	8.Was the scientific quality of the included studies used appropriately in formulating conclusions	9.Were the methods used to combine the findings of studies appropriate	10.Was the likelihood of publication bias assessed	11.Was the conflict of interest stated
Bjelakovic, 2008	YES	YES	YES	YES	YES	YES	YES	YES	YES	YES	YES
Cai, 2016	NO	NO	YES	Can't answer	YES	YES	YES	YES	YES	YES	YES
Chen, 2013	NO	YES	YES	NO	YES	YES	YES	YES	YES	YES	YES
Deng, 2016	NO	NO	YES	NO	YES	YES	YES	YES	YES	YES	YES
Deng, 2021	NO	NO	NO	NO	YES	NO	NO	YES	YES	YES	YES
Du, 2017	NO	YES	YES	NO	YES	YES	YES	YES	YES	YES	YES
Du, 2020	NO	YES	YES	NO	YES	YES	YES	YES	YES	YES	YES
Fang, 2015	NO	YES	YES	NO	YES	YES	YES	YES	YES	YES	YES
Ferro, 2018	NO	NO	YES	NO	YES	YES	NO	YES	YES	YES	YES
Fonseca-Nunes, 2014	NO	NO	NO	NO	YES	YES	NO	YES	YES	NO	YES
Grosso, 2017	NO	YES	YES	NO	YES	YES	Can't answer	YES	YES	YES	YES
Guo, 2015	NO	NO	YES	NO	YES	YES	YES	YES	YES	YES	NO
Han, 2015	NO	YES	YES	NO	YES	YES	YES	YES	YES	YES	YES
Huang, 2017	NO	YES	YES	Can't answer	YES	YES	YES	YES	YES	YES	YES
Khayatzadeh, 2015	NO	YES	YES	NO	YES	YES	NO	YES	YES	YES	YES
Kim, 2019	NO	YES	YES	NO	YES	YES	YES	YES	YES	YES	YES
Kong, 2014	NO	NO	YES	NO	YES	YES	YES	NO	YES	YES	YES
Lam, 2013	NO	NO	NO	NO	NO	YES	NO	NO	YES	NO	YES

Li, 2014	NO	NO	YES	NO	YES	YES	YES	YES	YES	NO	NO
Li, 2014	NO	NO	YES	NO	YES	YES	YES	NO	YES	Can't answer	YES
Li, 2018	YES	NO	YES	NO	YES	YES	YES	YES	NO	YES	YES
Liang, 2019	NO	YES	YES	NO	YES	YES	YES	NO	NO	Can't answer	YES
Lu, 2017	NO	NO	YES	NO	YES	YES	NO	NO	YES	YES	YES
Luo, 2021	NO	NO	YES	NO	YES	YES	YES	YES	NO	YES	YES
Miao, 2021	YES	YES	YES	NO	YES	YES	YES	YES	YES	YES	YES
Moazzen, 2020	NO	YES	YES	NO	YES	YES	YES	YES	YES	YES	YES
Mocellin, 2017	NO	NO	YES	NO	YES	YES	YES	YES	YES	YES	NO
Pabalan, 2014	NO	NO	NO	NO	YES	YES	YES	YES	YES	YES	YES
Pelucchi, 2011	NO	NO	NO	NO	NO	NO	NO	NO	YES	NO	YES
Schwingshackl, 2017	YES	NO	YES	NO	YES	YES	NO	NO	YES	YES	YES
Song, 2015	NO	YES	YES	NO	YES	YES	YES	YES	YES	YES	YES
Tio, 2014	NO	NO	YES	NO	YES	YES	NO	YES	YES	YES	YES
Tse, 2014	NO	NO	YES	NO	YES	NO	NO	YES	YES	YES	YES
Turati, 2015	NO	NO	NO	NO	YES	YES	NO	YES	Can't answer	YES	YES
Vingeliene, 2016	YES	NO	NO	NO	YES	YES	NO	YES	YES	YES	YES
Wang, 2014	NO	YES	YES	NO	YES	YES	YES	YES	YES	YES	YES
Wang, 2020	NO	NO	YES	Can't answer	YES	YES	YES	YES	YES	YES	YES
Wang, 2021	YES	NO	YES	Can't answer	YES	YES	YES	YES	YES	YES	NO
Wu, 2011	NO	YES	NO	NO	YES	YES	NO	YES	Can't answer	YES	YES
Wu, 2013	NO	NO	YES	NO	YES	YES	YES	YES	YES	YES	YES
Wu, 2015	NO	NO	YES	NO	YES	YES	YES	YES	YES	YES	YES
Yang, 2013	NO	NO	YES	NO	NO	YES	NO	YES	YES	YES	NO
Ye, 2017	NO	NO	YES	NO	YES	YES	YES	YES	Can't answer	YES	YES

Zhang, 2013	NO	YES	YES	NO	YES	YES	YES	YES	YES	YES	YES
Zhang, 2020	NO	YES	NO	NO	YES	YES	YES	YES	YES	Can't answer	YES
Zhang, 2020	NO	YES	YES	NO	YES	YES	YES	YES	YES	YES	YES
Zhang, 2020	NO	NO	NO	NO	YES	YES	YES	YES	YES	NO	YES
Zhao, 2017	NO	YES	YES	NO	YES	YES	YES	YES	YES	YES	YES
Zhou, 2016	NO	NO	YES	Can't answer	YES	YES	YES	YES	YES	Can't answer	YES
